# One Metre Plus (1M+): A Multifunctional Open-Source Sensor for Bicycles Based on Raspberry Pi

**DOI:** 10.3390/s21175812

**Published:** 2021-08-29

**Authors:** Andres Henao, Philippe Apparicio, David Maignan

**Affiliations:** Institut National de la Recherche Scientifique (INRS), Centre Urbanisation Culture Société, 385 Sherbrooke E, Montréal, QC H2X 1E3, Canada; carlosa.henaof@inrs.ca (A.H.); david.maignan@inrs.ca (D.M.)

**Keywords:** instrumented bikes, cyclist safety, bicycle video recording, lateral passing distance, overtaking distance, sensors, raspberry pi

## Abstract

During the last decade, bicycles equipped with sensors became an essential tool for research, particularly for studies analyzing the lateral passing distance between motorized vehicles and bicycles. The objective of this article is to describe a low-cost open-source sensor called *one metre plus* (1m+) capable of measuring lateral passing distance, registering the geographical position of the cyclist, and video-recording the trip. The plans, codes, and schematic design are open and therefore easily accessible for the scientific community. This study describes in detail the conceptualization process, the characteristics of the device, and the materials from which they are made. The study also provides an evaluation of the product and describes the sensor’s functionalities and its field of application. The objective of this project is to democratize research and develop a platform/participative project that offers tools to researchers worldwide, in order to standardize knowledge sharing and facilitate the comparability of results in various contexts.

## 1. Introduction

Over the past decade, many naturalistic cycling studies (i.e., mobile data collection) were undertaken by different researchers throughout the world. This stream of studies is mostly based on bicycles transformed into actual mobile laboratories, based on the use of a vast array of sensors [1]. These instrumented bicycles have become an essential tool for research. They allow us to understand cyclists’ behaviour in varying environments and evaluate their safety [1,2].

Among the studies using instrumented bicycles, there are usually five types: (1) studies on e-bikes to compare electric and traditional bicycles, in terms of speed, acceleration, and deceleration [3,4,5,6,7,8,9], (2) studies on cyclists’ exposure to air pollution and noise exposure [10,11,12,13,14,15,16,17], (3) studies on cyclist conflicts [18,19,20,21,22,23], (4) studies on vehicle detection when encountered met during trips [24,25,26], and finally, (5) studies on lateral passing distance by motorized vehicles [27,28,29,30,31,32,33,34].

In this article, we are particularly interested in this last stream, aiming mainly to identify dangerous overtaking (ex., less than one metre). In order to do so, measuring instruments allow us to determine the lateral overtaking distance with lidar or sonar distance sensors [27,28,29,30], establish the types of vehicles involved in an overtaking manoeuvre based on the video [27,28,29,31,32], verify the presence of parked vehicles on the road with the videos [27], measure the cyclist’s speed with the speedometer or the GPS [28,33,34], determine the geographical position during overtaking using the GPS [27,28,31,33,34], and measure the speed of the vehicles with speed sensors [31,33].

The goal of this work is to describe a low-cost open-source sensor adapted to the bicycle and allowing to measure mainly the lateral passing distance, but also the geographical position as well as record a video of the trip. The project was designated as being 1m+ or one metre plus. Since access to the plans and codes of the device are free for the scientific community in general, this project is part of the philosophy of Open-source Product Development (OSPD) [35,36,37]. Yet, compared to the use of open-source software (ex., OSGIS) and open geospatial data, OSPD is something seldom mobilized in urban studies and more specifically in the field of road safety studies [38,39].

The rest of the paper is organized as follows: a literature review of the instrumented bicycles used in passing distance studies, the materials used for the open-source sensor and the assembly characteristics, the product evaluation results, discussion and conclusion.

## 2. Review of Instrumented Bicycles for Measuring Lateral Distance Passing

### 2.1. Why Develop an Open-Source Product?

The increasing use of mobile data collection is due to many factors: the production of more compact sensors, the development of open-source hardware and software (microcontroller board and embedded single board computers), and new techniques for rapid prototyping (CNC, laser cut, and 3D printing), thus facilitating the development of custom-made products [1]. Nevertheless, conducting a naturalistic cycling study with an instrumented bike still presents four major challenges.

First, most products are not specifically designed for mobile data collection. Therefore, researchers must modify and adapt the bicycle frame devices, so that they are correctly positioned, usually by attaching them to the handlebars, or on the rear or front rack.

Second, few products include multiple functions. Therefore, researchers must use different devices for specific tasks such as distance sensors, GPS, cameras, vehicle speed sensors, pollution and noise sensors, etc. This contributes to a considerable increase in research costs.

Third, the plethora of configurations and devices used present a challenge for the merging and interoperability of data, since these are recorded in different formats and temporal resolutions depending on the sensors. It therefore becomes difficult to compare empirical results between the various studies.

Fourth, if they resort to commercial products (GPS, camera, distance sensor, etc.), researchers are limited to the functions and characteristics of the sensors as provided by the manufacturers, and these do not always adequately meet the needs for their studies.

To overcome these challenges, many researchers decided to develop custom-made products. However, these products are unavailable commercially, or the plans and development codes are not open. This therefore has a detrimental effect on the replicability of naturalistic cycling studies.

### 2.2. Devices for Measuring Lateral Passing Distance: A Brief Overview

Recent studies on lateral passing distance (LPD) are based on three types of approaches (Table 1). First, pioneer works on LPD were entirely conducted solely with cameras, that is, without resorting to distance sensor and GPS devices [29,40]. The LPD was evaluated by using footage mark over the video, which is an interesting approach but not very precise.

Second, many studies used a multitude of existing devices, particularly distance sensors, GPS, front and lateral cameras, and in some cases, speedometers, accelerometers, gyroscopes, and lasers [28,29,31,33]. Although this approach is interesting, it has two major drawbacks. On the one hand, these devices are not merged in the same unit. On the other hand, the total cost of the various devices is often quite onerous (between $1290 and $5980 US) (Table 1).

Third, the latest trend in studies is based on custom-made devices. Walker et al. [30] Used an MB1200 XL-MaxSonar-EZ0 distance sensor (Maxbotix Inc., Brainerd, MN, USA) connected to an Arduino Uno microcontroller card (Arduino, Scarmagno, Italy). The card registered the sensor’s distance captures by attributing time and date. The various elements, as well as the batteries, were assembled in a grey plastic box on the bicycle’s rear luggage carrier. In the case of Mehta et al. [34], the researchers designed a sensor with a microcontroller, a GPS, and a sonar-type distance sensor (no specifications for devices in the article). The devices were integrated into a sealed plastic grey box at the rear of the bicycle. The researchers also used a camera on the handlebars to observe the side of the cyclist. Finally, Beck et al. [27] designed a device called Metrebox. The device integrated a GPS (Adafruit Ultimate Feath-erWing, Adafruit Industries, New York, NY, USA), a distance sensor (MB1230 XL-MaxSonar-EZ3, Maxbotix Inc., Brainerd, MN, USA), and rechargeable lithium 18,650 batteries. The Metrebox used a box printed in 3D in which the devices are assembled. The researchers also used a camera (GoPro Hero5 Session, GoPro, San Mateo, CA, USA) attached to the handlebars to record the trips. As for the studies based on multiple existing devices, the set-up costs of custom-made devices is considerably less expensive (Table 1).

To our knowledge, to date, there exists only one commercial solution to measure LPD: the Codaxus C3FT v3 (Codaxus, Austin, TX, USA), at a cost of $1460 USD. This device uses a MB7066 XL-MaxSonar-WRL1 distance sensor (Maxbotix Inc., Brainerd, MN, USA). The device sends back distances between 0 to 250 cm with a frequency of 10 Hz. This device’s functions are quite limited since it excludes a GPS, camera, and especially, there is no data recording device.

## 3. Material and Methods

### 3.1. Preliminary Assessment

A preliminary analysis of the products was conducted to assess the feasibility of the project and identify possible technical solutions for an OSPD product. Two main findings emerge. First, many researchers use rapid prototyping techniques (3D printers and laser cut) to develop personalized cases [27,41]. Second, many of them use microcontrollers (ex., Arduino) [27,41] or single board nano computers (ex., Raspberry pi) [42] for data management.

Following the production of many prototypes, we decided to develop a product based on the Raspberry pi zero w (Raspberry Pi Foundation, Cambridge, UK). Many reasons motivated the choice of this nano computer, namely excellent online support, high-level connectivity with various devices (especially the connection of a video camera), size (65 mm × 31 mm × 5 mm), and the price between $10 and $25 US. As for the case, we decided to resort to a 3D printer, providing more flexibility for design and different prototypes.

### 3.2. Electronic Devices

An extensive search for available devices was conducted on the three most important dimensions for the analysis of LPD studies: detection of lateral distance, recording of geographical coordinates, and video recording. The selection of devices was also based on compatibility with Raspberry pi zero w, resolution (cost-benefit ratio), and size.

The final selection of devices is reported in Table 2. The distance sensor chosen for the project is the Tfmini plus micro lidar (Benewake, Beijing, China), capable of measuring up to 12 m and achieving up to 100 distance captures per second with an accuracy of ±5 cm at distances of less than 5 m. It is also possible to configure the capture cycle. Compared to devices used in previous studies (Table 1), the Tfmini plus micro lidar counts with superior capture frequency and distance measurement.

The GPS BN-220 (Beitian Shenzhen, Hong Kong, China) was selected for its work frequency of one cycle per second that can be modified, its spatial precision (two metres horizontally), and its adequate documentation and online support.

Usually, in studies analyzing overtaking distance, two cameras are used: a front camera to characterize the urban environment and a lateral camera to determine vehicle type. However, the Raspberry pi zero w can only manage one camera. For this reason, the fish-eye type cameras were privileged to allow for recording both front and side of the cyclist at the same time. We selected the Rpi Camera G (Waveshare, Shenzhen, Hong Kong, China) with a working angle of 160 degrees. It is also equipped with an infrared system for night vision and an adjustable resolution up to full HD (1920 × 1080 px).

In the following section, there are detailed descriptions of electrical connections and assembly of the device.

### 3.3. Connection Diagram

**Figure 1:**
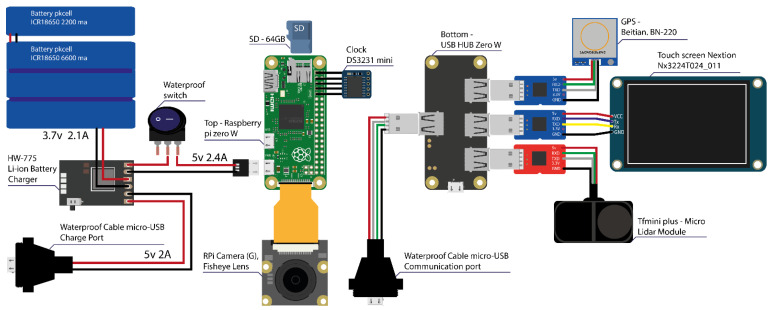
Schematic connection diagram.

The diagram of the connections between devices is presented in Figure 1. Three systems configure the device. First, the system to charge and manage the battery is composed of four elements:

- An HW 775 (Makerfocus, China) card which manages charging and discharging of the device and also protects the system from eventual electrical peaks.
- Four lithium Pkcell batteries (Shenzhen, Hong Kong, China) of 2200 mAh each for a total of 8800 mAh (approximately seven hours of continuous recording).
- A sealed Twidec switch (Suzhou, China) to allow the current to be conducted to the device as a whole.
- A sealed micro Cerrxian USB port (China) to recharge the batteries and to which it is also possible to connect Power Banks to increase autonomy during a data collection session.

The second system is responsible for data recording and device management: the Raspberry pi zero w with an SD card of 64 GB to record trips between 15 and 30 h according to camera resolution.

**Figure 2:**
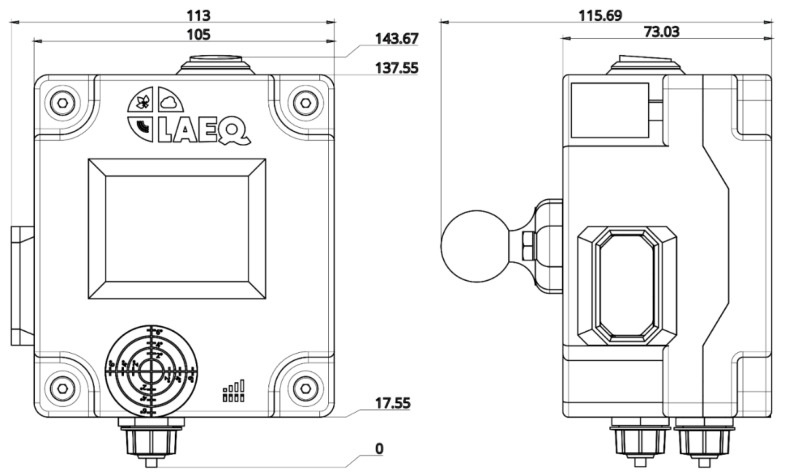
One metre plus (1m+) sensor, distances in mm.

The last one is the peripheral management system. The GPS, distance sensor, and screen use series communication protocols, while the camera uses an MIPI camera serial interface (CSI). The camera is directly connected to the CSI port of the Raspberry pi zero w (Figure 2).

Other elements are necessary to guarantee the proper operation of the device:

- The tactile screen as a collection device for user commands.
- The DS3231 RTC clock which memorizes the time and transmits it to the Raspberry pi, the latter being directly welded to the superior pins of the nano computer.
- Finally, the hub zero USB is the connection bridge between the devices from 3 USB adaptors toward ttl, one last USB micro port is available to download the collected files during the bicycle trips.

### 3.4. Software

The operating system used on the Raspberry pi zero w is the Raspberry Pi OS (32 bit) based on Debian [43]. The programming language selected for the development of the project was Python (version 3.7.3) [44], using many libraries: pyserial (3.5), multiprocess (0.70.11.1), raspivid camera app, path (15.1.2), pynmea2 (1.18.0), and PyYAML (5.4.1). The source codes of one metre plus software are available free of charge (under the GPLv3 licence) on GitHub [45].

Three types of files are obtained through the data collection process:

- For the distance sensor, a CSV file including the time (in milliseconds) of the event and the distance in centimetres.
- For the GPS, a CSV file with the time (in milliseconds) and the coordinates of the geographical position (longitude and latitude).
- For the fish-eye camera, a file of h.264 format.

These three files were recorded with the identification of the device, the date and time of the beginning of the recording (e.g., ID1_C1_2021_05_20_20_14_08_15.h264).

### 3.5. Product Design and 3D Modeling

Since the one meter plus sensor is based on the integration of the various devices described previously, there are no available cases on the market for the assembly of the different components. Therefore, we have created a model for a personalized 3D case for the 1m+. The 3D Modelling was done on Onshape [46]. Figure 2 shows the dimensions of the 1m+ sensor (for more details on parts, assembly, and devices, see Supplementary Material, S1–S3).

The different elements (Table 3) were printed on the Creality 3D CR-10s printer (Creality3D, Dalang, Shenzhen, China). The printing characteristics are the following: Layer height of 0.2 mm, infill density of 70%, wall lines count of 3 and support on. The printing material used was the white PLA (Polylactic Acid). Total duration of 3D printing of a 1m+ sensor is 51 h (with Creality 3D CR-10s printer) and approximately 380 g of PLA are necessary for its production. The 3D models and plans are available free of charge licensed under the Creative Commons Attribution-NonCommercial 4.0 International Licence (CC-BY-NC).

**Figure 3:**
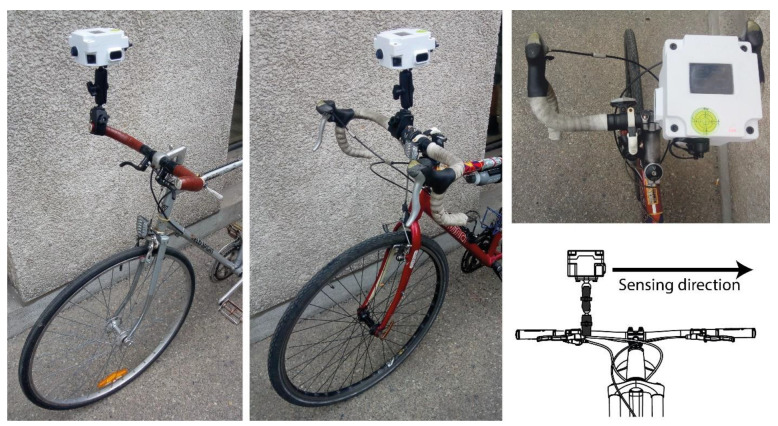
Integration of the one metre plus sensor to the bicycles.

The 1m+ sensor includes a diamond baseball-type inferior support (RAM^®^ B 238U, Seattle, WA, USA); this physical element, as well as the double socket arm (RAM^®^ B 400U, Seattle, WA, USA) and the tough claw (RAM^®^ B 201U, Seattle, WA, USA), greatly facilitate the attachment of the sensor to the bicycle. The three elements facilitate the attachment of the sensor in many positions and diameters of handlebars (Figure 3). An important characteristic of the mobile data collection is the horizontal position of the camera; to guarantee this condition, the 1m+ device has a spirit level on the top cover.

The final price of the product is approximately 500 US$ (values may vary according to the availability of products); this value includes electronic materials, screws, and various elements for assembly, the RAM^®^’s supports and the printed plastic case. The value does not include assembly costs. The assembly and installation time are about 6 hours. For more details on the list of materials, see the Supplementary Material (S2).

### 3.6. User Interface

**Figure 4:**
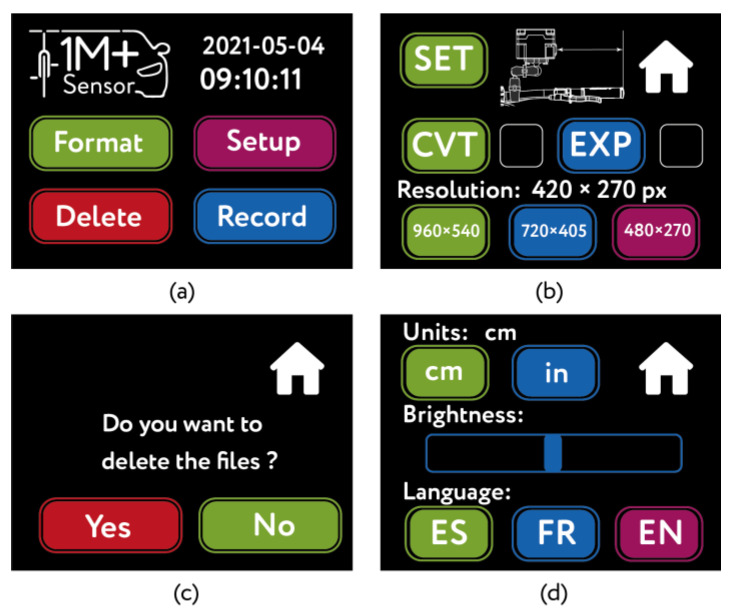
Graphical user interface. (**a**) main page; (**b**) Configuration page activated by the button Setup. (**c**) Delete page activated by the button Delete. (**d**) Format page activated by the button Format.

**Figure 5:**
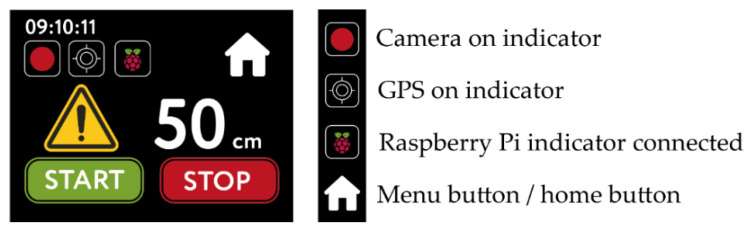
Recording page.

To manipulate the 1m+ sensor, the tactile screen includes a specific editor (nextion-editor v1.631, Nextion, Shenzhen, Hong Kong, China) to implement and develop the graphic interface. The interface is composed of the main menu and the format, configuration, file removal, and recording pages (Figure 4 and Figure 5).

The main menu shows time and date, as well as the buttons granting access to the pages (Figure 4a).

On the configuration page, the user may regulate the distance in relation to the edge of the handlebar for the data collection process (Figure 4b). Still on the same page, two buttons are available for file management and exportation: the *CVT button* (Convert) converts the video files from the h264 format (native to the camera) to the mp4 format; the *EXP button* (Export) exports files recorded by the device on a USB key. The user may also define the camera resolution (960 × 540, 720 × 405 and 480 × 270 px).

The deletion page allows the user to eliminate files recorded by the sensor (Figure 4c). In the *Format* page, the user can define the units of measure (metric or imperial), modify the brightness of the screen, and choose between three languages (Spanish, English or French) (Figure 4d). On each page, there is a *Home* button to return to the main menu.

The last page is the recording interface (Record button). In the top left corner, time is indicated. Once the user clicks on the start button (START), the system launches the respective commands to begin data recording. Three icons indicate the status of the device to the user: one for the beginning of the video recording, a second one for the status of connection of the GPS, and the last one for the connection of the Raspberry pi zero w (Figure 5). As for distance, the numerical indicator shows the respective values with a resolution of 1 cm; if the value measured by the sensor is inferior to 100 cm, a danger icon appears at the left of the distance value. Finally, the *stop button* (STOP) finalizes the recording process.

### 3.7. Data Collection

To evaluate the 1m+ sensor, data collection was conducted in Montréal (Canada), including the first two authors on 8 June 2021, from 8:00–10:30 a.m. The cyclists covered 30 km individually. Each bicycle was equipped with a 1m+ sensor, the referral distance at the edge of the handlebars was determined at the beginning of the collection. The participants had to travel the same circuit twice with different camera resolutions: during the first lap, cyclists travelled with the highest resolution (960 × 540 px) and, for the second lap, one participant cycled with medium resolution (720 × 405 px), and the other with minimal resolution (480 × 270 px). The two sensors were pre-calibrated at two metres using a flexometer. The study was conducted in accordance with the Declaration of Helsinki, and the protocol was approved by the Ethics Committee of Institut national de la recherche scientifique (project No. CER 19-509).

**Figure 6:**
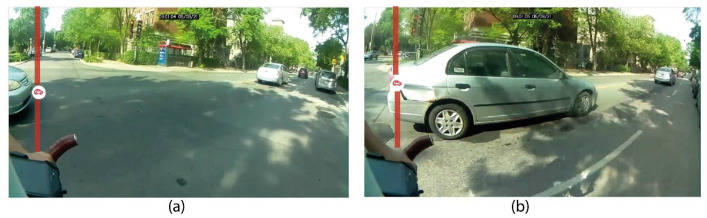
Characterization of overtakings on Vifeco. (**a**) beginning of overtaking aligned with cyclist’s handlebars; (**b**) end of overtaking.

The structuring of data was completed with version 4.0.2 of the R software [47]. Since the sensor can measure distance for an array of objects, it is therefore relevant to identify the values generated by vehicle overtakings. Consequently, the tracking of these overtakings was conducted with a visual analysis of videos with the Vifeco free software [48] and starting with a counting guide (presented in the Supplementary Material, S4). According to this guide, it is necessary to situate an observation at the beginning of the overtaking (front end of vehicle aligned with the cyclist’s handlebars, Figure 6a), and a second observation at the end of the overtaking (extremity of the vehicle aligned with the cyclist’s handlebars, Figure 6b).

The JSON files obtained from Vifeco were imported in the R software with the beginning and the end of each overtaking (in milliseconds). Once paired (timestamp) with data for one metre plus distances, it is then possible to obtain the minimal distance during overtaking. Subsequently, minimal distance values for each overtaking were combined with the geographical position according to the time associated with GPS coordinates.

## 4. Results

During the trips for this test collection, the two 1m+ sensors recorded close to four hours of travel, corresponding to approximately 12 GB (for both sensors); no loss of data was found. The files obtained by the two sensors are presented in detail in Table 4. As expected, camera resolution had an important impact on the size of the MP4 output file.

### 4.1. Spatial Observations

**Figure 7:**
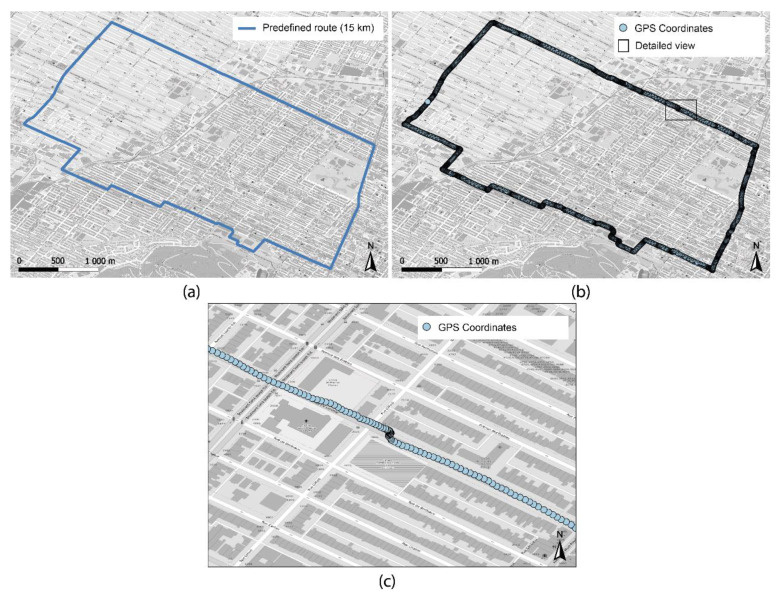
Characteristics of the trip (**a**) predefined trip; (**b**) data collected by the 1m+ sensor; (**c**) detailed view of data collected by the 1m+ sensor.

As for the GPS coordinates, Figure 7a presents the predefined trip for product testing. Figure 7b shows one of the four tracks of spatial coordinates obtained by the 1m+ sensor. Each point/observation/coordinate is one second apart, therefore, responding to the technical characteristics of the device. The adequate precision of the GPS and the low distribution of observations must be noted (Figure 7c).

**Figure 8:**
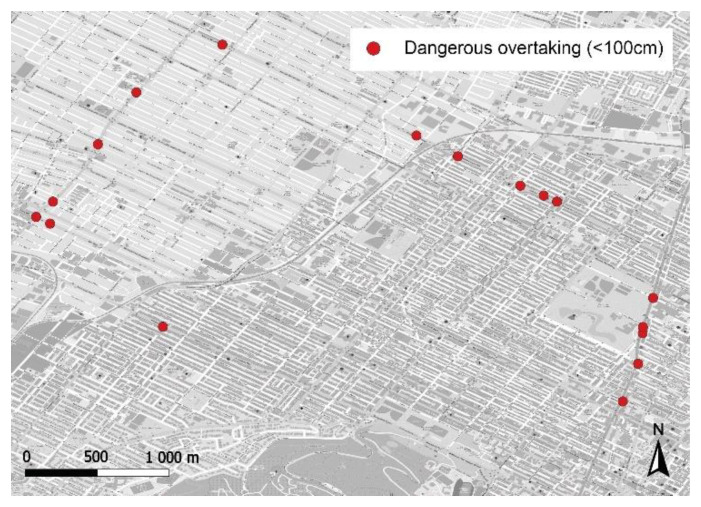
Data collected with dangerous overtakings (less than 100 cm).

Figure 8 shows 17 locations where cyclists experienced a dangerous overtaking, that is with a lateral passing distance less than one metre.

### 4.2. Camera Resolution

**Figure 9:**
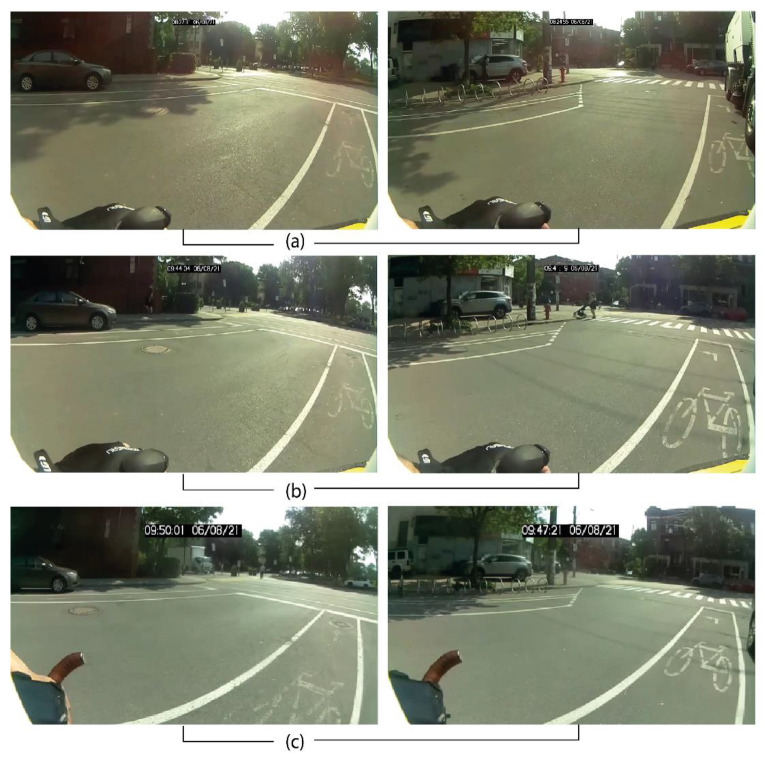
Different resolutions of the 1m+ camera sensor (**a**) 920 × 540 px (**b**) 720 × 405 px (**c**) 480 × 270 px.

Figure 9 is constructed with images at two different intersections extracted from the videos recorded by the 1m+ sensors and with three available resolutions: 920 × 540 px (Figure 9a) 720 × 405 px (Figure 9b) et 480 × 270 px (Figure 9c).

### 4.3. Distance Measures by Vehicle

**Figure 10:**
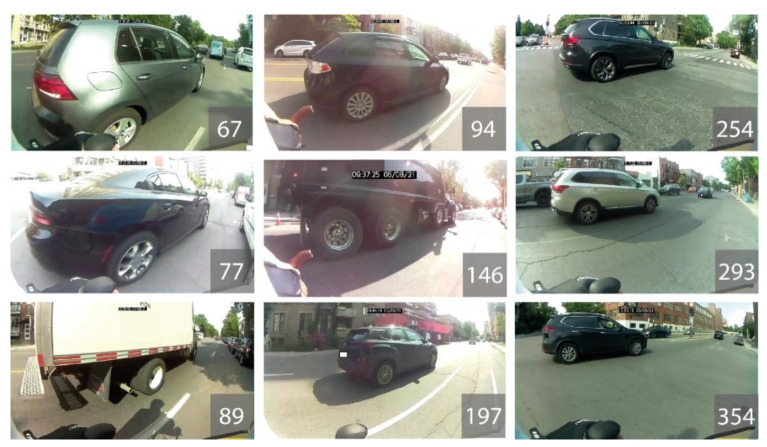
Overtaking by vehicles according to minimal distance in centimetres.

A total of 317 overtaking manoeuvres were recorded; the minimal distance was 67 cm and the maximal distance was 354 cm. Figure 10 illustrates different values of lateral passing distance with the image of the vehicle having performed the manoeuvre.

### 4.4. Technical Data—Product Characteristics

The technical specifications of the product summarize the functions and characteristics described previously (Table 5).

## 5. Discussion

### 5.1. Comparison with Previous Devices

Table 6 presents a summary of the characteristics of custom-made devices used to analyze cyclists’ safety based on LPD. First, most of them have a distance sensor, but a 1m+ sensor presents a capture cycle per second six times higher (60 Hz versus 10 Hz). We must remember that our device’s capture cycle can be reduced in the open Python code. Second, the range distance is larger and adjustable as well (0.1 to 12 m). Usually, overtakings of more than four metres are seldom considered in studies. Again, the distance threshold can be modified in the settings file.

Third, the GPS records one observation per second as do most devices. This capture cycle can require a lot of energy. Consequently, this value can be increased with the device (ex., one observation every five seconds) in cases where high accuracy of a trajectory is not necessary. In such cases, using post-treatment map-matching algorithms would be recommended such as that proposed by Li et al. [49] to improve the GPS tracking.

Fourth, the camera resolution is less than the Go Pro hero 5 session but based on our experience, a resolution of 426 × 240 px is sufficient to conduct an analysis of environmental characteristics. As a comparison, the camera used in the 1m+ costs about $25 US, versus $400 US for the Go Pro hero 5 session [27].

Finally, most of the custom-made products can be manufactured through rapid prototyping technologies; like Beck et al. [27], we opted for 3D printing. The most significant distinction of our device is access to 3D plans and templates. Also, 1m+ sensor fits with the OSPD philosophy, users being able to utilize it, modify it, and share it.

Unfortunately, an evaluation/comparison of the devices in real conditions is not possible because the plans, codes, and materials of other devices are unavailable online.

### 5.2. Limits of the Sensor and Potential Improvements

During the analysis of the videos to identify overtaking manoeuvres, we found a significant number of vibrations on the videos for certain sections of the trip. This factor varies according to bicycle suspension, road condition, cyclist’s driving style, among others. Therefore, future versions of the 1m+ sensor should include physical elements that reduce vibrations to improve the quality of the videos and increase the sensor’s life span (by reducing the impacts). It is also possible to reduce video vibrations in postprocessing, with libraries such as vidstab [50] or opencv [51]. Once data are collected, two avenues could be explored in future works: automatic detection of passing vehicles by using Yolo [24,25,26], and improvement of the map-matching process. On this last point, each location of the GPS coordinates could be matched with an image extracted for the video (recorded by the wide-angle camera) to assess spatial accuracy and modify the location when needed.

This version of 1m+ is only dedicated to identifying dangerous overtakings. However, other elements could be added in future versions to characterize the cyclist’s behaviour and their safety during a route:

- A microphone to record the video with the environmental sound.
- A Bluetooth speedometer (with a sensor attached to the wheel) to accurately measure speed and distance.
- Gyroscopes and accelerometers to measure cyclist stability during the overtaking manoeuvre.
- A push-button that allows the cyclist to identify dangerous manoeuvres or other conflicts. This could be useful to evaluate differences between perceived and real risks.

It would also be practicable to create ecosystems between these different devices with Wi-Fi or Bluetooth connectivity.

### 5.3. Contribution to Research

Considering the results of the device, we believe that the 1m+ sensor can become an essential element in research with equipped bicycles. Free access to the plans and software are elements that can motivate other researchers to use the product and/or create new versions that can be adapted to their needs. Although the 1m+ sensor is initially meant for LPD analysis, some of its functions could be useful in cyclist conflicts studies, the detection of vehicles, and studies with e-bikes. Based on the data collected by the 1m+ combined with video analysis, it is possible to evaluate the influence of various environmental factors on the safety of cyclists.

## 6. Conclusions

In this study, we described an open-source sensor capable of measuring vehicle overtaking distances, recording geographical location and a video of the trip. Among the comparable advantages of the 1m+ in relation to other devices, let us mention others: the integration of many functions in the same device, facility in data management (all devices are synchronized with the same temporal reference), access to variables by default to modify the parameters of the device, and access to the 3D model for purposes of modification, if necessary.

The implementation of this project is mainly oriented by the democratization of a research tool on the safety of cyclists, by proposing a free and participative platform/product. This device could therefore be used by different members of the academic community or organizations interested in dangerous overtaking of cyclists in a given city. Also, police services in many cities and countries around the world could use the 1m+ sensor to evaluate whether the legislation in effect on overtaking distances with cyclists is respected.

## Figures and Tables

**Table 1:** Summary of instrumented bikes by devices in studies on lateral passing distance.

	Distance Sensor			GPS		Camera		Product	Product
Study (Year)	Product	Hz	Range Distance (m)	Product	Hz	Product	Resolution(px)	Additional Device	Type and Cost ($US) of the Final Product
Parkin et al. (2010) [32]	NA	NA	NA	NA	NA	Helmet Camcorder Generation 5 (Archos, Igny, France)	640 × 480	Archos 605 MP4 recording device (Archos, Igny, France)	Multiple existing devices. 600 $US
Stewart et McHale (2014) [40]	NA	NA	NA	NA	NA	AT1 and ATC5K Waterproof Action Camera (Oregon Scientific, Portland, OR, USA)	640 × 480	NA	Multiple existing devices. 150 $US
Shackel et al. (2014) [29]	M-300/95 Sensor (Massa, Hingham, MA, USA)	95,000	0.3–4	NA	NA	Viosport POV 1.5 camera × 2 (V.I.O, New Hope, PA, USA)	720 × 480 (30 fps)	Laser pointer for distance from the kerb	Multiple existing devices.Price: NA
Chuang et al. (2013) [28]	MB1200 XL-MaxSonar-EZ0 × 2 (Maxbotix Inc., Brainerd, MN, USA)	60–70	0–6.5	GPS (Canmore Electronics Co Ltd. Jhubei, Hsin Chu, Taiwan)	1	Car camera DVR black box × 5 (DOD Tech Co Ltd., Taoyuan, Taiwan)	860 × 640 (30 fps)	Multi-function logger PhidgetSpatial Precision 3/3/3 (Phidgets Inc., Calgary, AB, Canada)	Multiple existing devices. 1290 $US
Dozza et al. (2016) [31]	LIDAR system UXM-30LXH-EWA (Hokuyo, Osaka, Japan)	20	ND	ND	1	ND	1920 × 1080 (30 fps)	NA	Multiple existing devices. 5980 $US
Llorca et al. (2017) [33]	TruSense S200 Laser Sensor × 2 (Laser Technology Inc., Centennial, CO, USA)	55,000	0.5–750	Video VBOX waterproof 10 HZ gps data logger (Racelogic, Buckingham, UK)	10	Video VBOX waterproof 10 HZ gps data logger (Racelogic, Buckingham, UK) with 3 cameras	ND	TrueSense T100 (Laser Technology Inc., Centennial, CO, USA) for measure the speed of overtaking vehicles.	Multiple existing devices. 3695 $US
Walker et al. (2014) [30]	MB1200 XL-MaxSonar-EZ0 (Maxbotix Inc., Brainerd, MN, USA)	10	ND	NA	NA	NA	NA	Arduino Uno (Arduino, Scarmagno, Italy)	Custom-made. 120 $US
Mehta et al. (2015) [34]	ND	10	0.3–4.8	ND	ND	Camera lateral (type not defined)	ND	NA	Custom-made. Price: NA
Beck et al. (2019) [27]	MB1230 XL-MaxSonar-EZ3 (Maxbotix Inc., Brainerd, MN, USA)	10	0–3.3	Adafruit Ultimate GPS FeatherWing (Adafruit Industries, New York, NY, USA)	1	GoPro Hero 5 Session (GoPro, CA, USA) in the handlebar	ND	NA	Custom-made. Price: 693 $US

ND: Not defined, NA: Not applicable. The cost (in dollars) is estimated by summing the price of each electronic device.

**Table 2:** Summary of electronic devices for the sensor one meter plus (1m+).

Device	Model	Manufacturer	Specifications	Quantity	Price (US)
Screen touch	NX3224T024	Nextion (Shenzhen, Hong Kong, China)	Resolution: 320 × 240 px	1	30
			Color: 65,536 colors		
			Voltage: 5 V		
Distance sensor	Tfmini plus micro lidar	Benewake (Beijing, China)	Range: 0.1–12 m	1	60
			Frequency: 100 Hz		
			Resolution: 1 cm		
			Accuracy: ±5 cm (0.1–5 m); ±1% (5–12 m)		
			Voltage: 5 V		
GPS	BN-220	Beitian (Shenzhen, Hong Kong, China)	Frequency: 1 Hz	1	16
			Accuracy: 2 m in horizontal position		
			Voltage: 5 V		
Small single board computer	Raspberry Pi Zero W	Raspberry Pi Foundation (Cambridge, UK)	Memory: 512 MB RAM	1	30
			Connectivity: Bluetooth and Wifi		
			Processor: 1 GHz single-core CPU		
			Voltage: 3.3 V		
Hub usb	Hub zero w-BH10128PSU	Makerspot	Socket type: 4 port USB	1	17
Camera	RPi Camera G	Waveshare (Shenzhen, Hong Kong, China)	Field of view: 160 degrees	1	25
			Sensor resolution: Max 1080 p		
			Aperture (F): 2.35		
			Voltage: 3.3 V		
Battery Charger	HW-775	Makerfocus (China)	Charging current: 0–2.1 A	1	15
			Discharge current: 0–2.4 A		
			Input voltage: 5 V		
			Output voltage: 3.7–5 V		
Waterproof Cable micro-USB	Micro USB mount extension	Cerrxian (China)	Connector: Micro USB male/female	1	15
SD Card	EVO Select micro SDXC 64 Gb	Samsung (Seoul, South Korea)	Capacity: 64 GB	1	15
Clock	DS3231 Real time clock	Daoki (China)	Voltage: 3.3 V or 5 V	1	4
			time accuracy: ±0.4 s/day		
Waterproof switch	Round Rocker Switch Blue	Twidec (Suzhou, China)	Voltage: 12 V	1	3
			Current: 20 A		
Battery	3.7 V Li-ion 18650	Pkcell (Shenzhen, Hong Kong, China)	Voltage: 3.7 V	4	22
			Nominal Capacity: 2200 mAh		
Usb to ttl connector	CP 2102	Izokee (China)	Output Voltage: 3.3 or 5 V	2	20
			Communication protocol: UART		
Usb to ttl connector	RS232	Robojax (China)	Output Voltage: 3.3 or 5 V	1	10
			Communication protocol: UART		
Total:					292

Note: This list only includes electronic devices.

**Table 3:** 3D parts specifications.

Element	Size (High, Width and Depth in mm)	Time of Printing	Material Quantity (g)
Top cover	105 × 120 × 34	15 h 40 min	128
Bottom case	113 × 122 × 62	28 h 43 min	213
Raspberry pi support	92 × 6 × 32	6 h 23 min	39
Distance sensor support	35 × 14 × 26.5		
Camera support	38 × 17.5 × 25		
Touch screen support	80 × 61 × 6		
Charger support	24 × 17 × 3		

**Table 4:** Files specifications.

File	Distance File	GPS File	Video File			
	Size (KB)	Size (KB)	Resolution (px)	Size H264 (KB)	Size MP4 (KB)	Duration (mm:ss)
ID1_C1_2021_06_08_08_14_39	841	156	960 × 540	4,027,668	4,028,034	52:36
ID1_C1_2021_06_08_09_29_01	633	154	720 × 405	3,226,752	3,227,105	52:06
ID2_C2_2021_06_08_08_19_49	1081	169	960 × 540	3,789,376	3,789,765	56:48
ID2_C2_2021_06_08_09_33_16	794	169	480 × 270	985,284	985,639	57:00

**Table 5:** Technical specifications one metre plus (1m+).

Charge		Distance Sensor	
Charging voltage	5 V	Range (adjustable)	2 cm–400 cm
Charging current	2 A	Capture frequency (adjustable)	30–40 Hz
**Battery**		**GPS**	
Capacity	8800 mAh	Horizontal resolution	2 m
Technology	Li-ion	Capture frequency (adjustable)	1 Hz
Operation voltage	3.7 V	**Global system**	
Operation current	1 A	Maximum operation time	7 h of continuous recording
**Camera**		Operating System	Raspberry Pi OS (32 bit)
Angle	160 degrees	Wifi	802.11 b/g/n
Resolution, by default Large (adjustable)	- Low (426 × 240 px) - Medium (768 × 432 px) - Large (960 × 540 px)	Bluetooth	v4.1 BLE standard
Aperture (F)	2.35	CPU	1 GHz single-core
Format	h.264 and mp4	RAM	512 MB
Framerate (adjustable)	25 fps	Spirit Level	Integrated

**Table 6:** Comparison between one metre plus (1m+) and other devices.

	Distance Sensor			GPS		Camera		Technology
Study (Year)	Feature	Hz	Range Distance (m)	Feature	Hz	Feature	Resolution	
Walker et al. (2014) [30]	√	10	ND	x	NA	x	NA	Custom made: Grey plastic box and commercial devices
Mehta et al. (2015) [34]	√	10	0.3–4.8	√	ND	√ (not integrated)	ND	Custom made: Grey plastic box and commercial devices
Beck et al. (2019) [27]	√	10	0.3–3.3	√	1	√ (not integrated)	3840 × 2160	Custom made: 3D printing and commercial devices
Codaxus C3FT v3	√	10	0–2.5	x	NA	x	NA	Standard product.
1m+ sensor	√	60	0.1–12	√	1	√	Max 1920 × 1080	Custom made: 3D printing and commercial devices

ND: Not defined, NA: Not applicable.

## Supplementary Materials

The following are available online at https://www.mdpi.com/article/10.3390/s21175812/s1, S1: Detailed list of products, S2: Plans, S3: Connection diagram, S4: Counting Guide.
