# Supplementary material for: One Metre Plus (1M+): A Multifunctional Open-Source Sensor for Bicycles Based on Raspberry Pi
Source: Sensors (Basel). 2021 Aug 29;21(17):5812. doi: 10.3390/s21175812 (PMC8433888; doi:10.3390/s21175812)
Supplement: Supplementary file 1 [file sensors-21-05812-s001.zip › sensors-1335368-supplementary.pdf]

Supplementary material for the article: One meter plus (1M+), a multifunctional open source sensor for bicycles based on raspberry pi.

1. Detailed list of products

Article:
Andres Henao (carlosa.henaof@inrs.ca)
Philippe Apparicio (philippe.apparicio@inrs.ca)
David Maignan (davidmaignan@gmail.com)

Hardware:
Andres Henao (carlosa.henaof@inrs.ca)

Software:
Andres Henao (carlosa.henaof@inrs.ca)
David Maignan (davidmaignan@gmail.com)

Supervisor:
Philippe Apparicio (philippe.apparicio@inrs.ca)

One meter plus (1M+)
A multifunctional open source sensor for bicycles based on raspberry pi.

Detailed list of products

Item	Quantity	Name	Manufacturer
1	1	Bottom case	3d printed
2	1	Top cover	3d printed
3	1	Distance sensor support	3d printed
4	1	Camera support	3d printed
5	1	Touch screen support	3d printed
6	1	Acrylic cover	3d printed
7	1	Raspberry pi support	3d printed
8	1	Charger support	3d printed
9	Standard products		
9.1	1	RAM® ball support diamond	RAM® B 238U (Seattle, Washington, USA)
9.2	1	RAM® double socket arm	RAM® B 400U (Seattle, Washington, USA)
9.3	1	RAM® tough claw	RAM® B 201U (Seattle, Washington, USA)
9.4	1	HW-775 Charger	Makerfocus (China)
9.5	1	SD Card 64 Gb	Samsung (Seoul, South Korea)
9.6	1	Bull eye level 32 mm	Taskar
9.7	1	Lithium Battery ICR 18650 x 3	Pkcell (Shenzhen, Hong Kong, China)
9.8	1	Lithium battery x ICR 18650 x 1	Pkcell (Shenzhen, Hong Kong, China)
9.9	2	Waterproof micro usb port	Cerrxian (China)
9.10	3	USB to ttl connectors cp2102	Izokee (China)
9.11	1	Waterproof switch Round Rocker Switch Blue	Twidec (Suzhou, China)
9.12	1	Raspberry pi zero w	Raspberry Pi Foundation (Cambridge, UK)
9.13	1	USB HUB Zero - BH10128PSU	Makerspot
9.14	1	Touch screen nextion NX3224T024	Nextion (Shenzhen, Hong Kong, China)
9.15	1	Raspberry Pi Camera (G) 160°	Waveshare (Shenzhen, Hong Kong, China)
9.16	1	Tfmini plus micro lidar	Benewake (Beijing, China)
9.17	1	Clock DS3231 RTC	Daoki (China)
9.18	1	GPS Beitian BN 220	Beitian (Shenzhen, Hong Kong, China
9.19	4	Hex socket head cap screw M5x0.8x20 mm	Local Supplier
9.20	4	Hex locking nut M5x0.8	Local Supplier
9.21	4	Hex socket head cap screw M5x0.8x20 mm	Local Supplier

One meter plus (1M+)
A multifunctional open source sensor for bicycles based on raspberry pi.

Detailed list of products

Item	Quantity	Name	Manufacturer
9.22	2	Hex socket head cap screw M6x1.0x20 mm	Local Supplier
9.23	2	Hex locking nut M6x1.0	Local Supplier
9.24	9	Hex socket head cap screw M3x0.5x 7 mm	Local Supplier
9.25	10	Hex socket head cap screw M2x0.4x7 mm	Local Supplier
9.26	10	Knurled Nut insert M2	Local Supplier
9.27	9	Knurled Nut insert M3	Local Supplier
9.28	4	Hex plastic standoff for raspberry pi M2.4x10mm+6mm	Makerspot
9.29	2	Micro USB Type B Male Connector	Uxcell
9.30	1	Standard USB Type A Male Connector	Biuzi
10	Other		
10.1	NA	Solder	
10.2	NA	24-28 AWG wires	
10.3	NA	Heat Shrink Tubing Cable	
10.4	1	Accessories Hub Micro USB OTG 4 ports	MakerSpot

NA : Not applicable

Supplementary material for the article: One meter plus (1M+), a multifunctional open source sensor for bicycles based on raspberry pi.

2. Plans

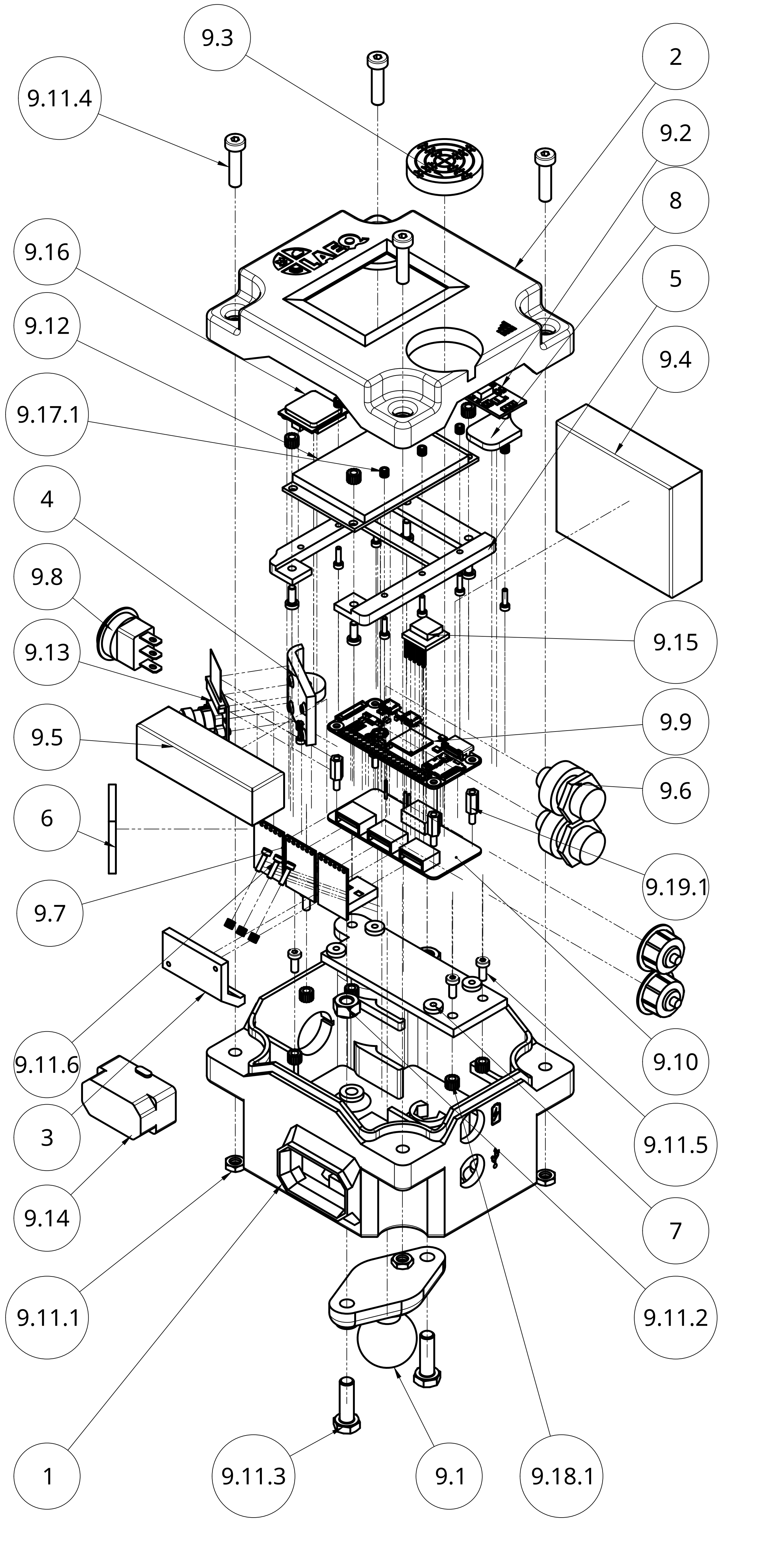

Item	Quantity	Name
1	1	Bottom case
2	1	Top cover
3	1	Distance sensor support
4	1	Camera support
5	1	Touch screen support
6	1	Acrylic cover
7	1	Raspberry pi support
8	1	Charger support
9	1	Standard products
9.1	1	RAM® ball support diamond
9.2	1	HW-775 Charger
9.3	1	Bull eye level
9.4	1	Lithium Battery x 3
9.5	1	Lithium battery x 1
9.6	1	Waterproof micro usb port
9.7	1	USB to ttl connectors
9.8	1	Waterproof switch
9.9	1	Raspberry pi zero w
9.10	1	USB HUB Zero
9.11	1	Nuts and screws
9.11.1	4	Hex thin nut grade A & B M5x0.8
9.11.2	2	Hex nut style 2 grade A & B M6x1.0
9.11.3	2	Hex head screw grade A & B M6x1.00 x 20
9.11.4	4	Hex socket head cap screw M5x0.80 x 20
9.11.5	9	Hex socket head cap screw M3x0.50 x 8
9.11.6	10	Hex socket head cap screw M2x0.40 x 8
9.12	1	Touch screen nextion
9.13	1	Raspberry Pi Camera (G) 160°
9.14	1	Tfmini plus micro lidar
9.15	1	Clock DS3231 RTC
9.16	1	GPS Beitian BN 220
9.17	1	Knurled nut insert M2
9.17.1	10	Knurled Nut Insert M2
9.18	1	Knurled nut insert M3
9.18.1	9	Knurled Nut Insert M3
9.19	1	Hex Standoff M2.5 x 10 mm
9.19.1	4	Hex standoff M2.5 x 10 + 6mm

UNLESS OTHERWISE SPECIFIED, DIMENSIONS ARE IN MILLIMETERS

Conception: Andres Henao
carlosa.henaof@inrs.ca
Programming:
Andres Henao
carlosa.henaof@inrs.ca
David Maignan
davidmaignan@gmail.com
Supervisor: Philippe Apparicio
philippe.apparicio@inrs.ca

	NAME	SIGNATURE	DATE
DRAWN	ANDRES HENAO		2021-05-13
CHECKED			
APPROVED			

MATERIAL

FINISH

One metre plus (1M+): a multifunctional open source sensor for bicycles based on raspberry pi

TITLE
One metre plus
Bill of materials

SIZE | DWG NO. | REV.

SCALE 1:2 | WEIGHT | SHEET 1 of 10

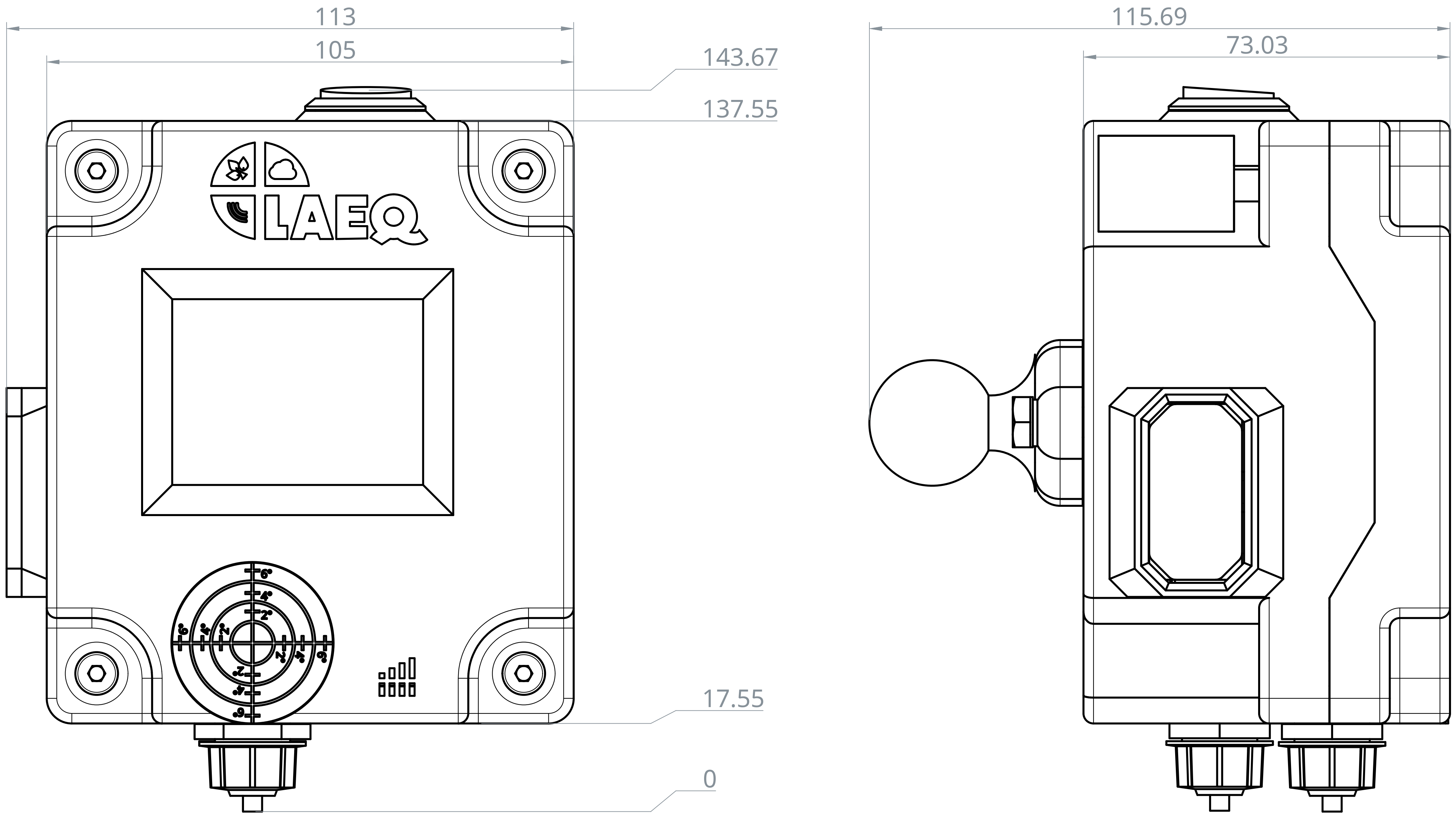

UNLESS OTHERWISE SPECIFIED, DIMENSIONS ARE IN MILLIMETERS		NAME	SIGNATURE	DATE
Conception: Andres Henao carlosa.henaof@inrs.ca	DRAWN	ANDRES HENAO		2021-05-13
Programming: Andres Henao carlosa.henaof@inrs.ca David Maignan davidmaignan@gmail.com	CHECKED			
	APPROVED			
Supervisor: Philippe Apparicio philippe.apparicio@inrs.ca	MATERIAL		FINISH	

One metre plus (1M+): a multifunctional open source sensor for bicycles based on raspberry pi

TITLE
One metre plus
Assembly

SIZE	DWG NO.	REV.
A3		

SCALE	WEIGHT	SHEET
1:1		2 of 10

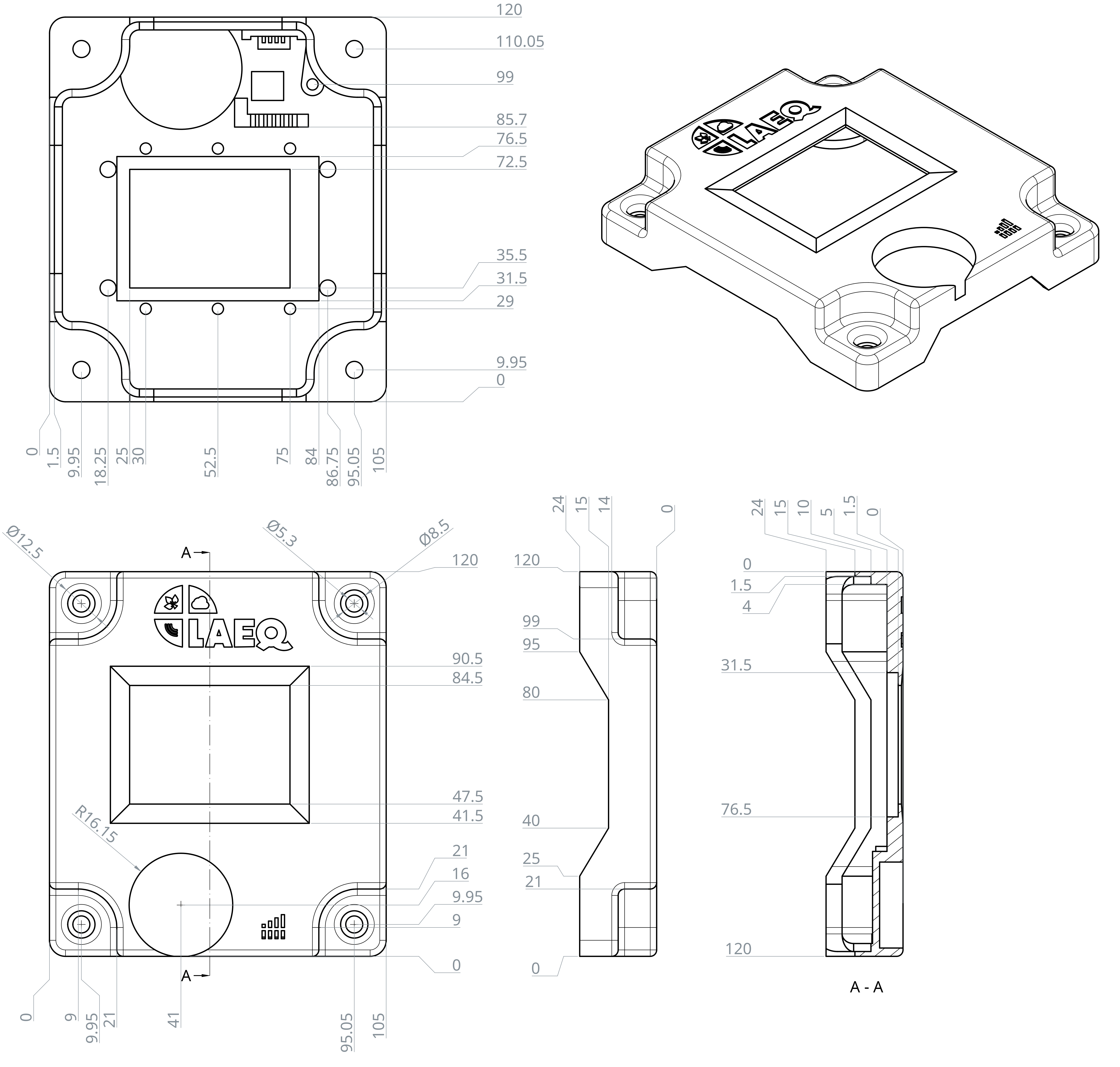

		NAME	SIGNATURE	DATE	One metre plus (1M+): a multifunctional open source sensor for bicycles based on raspberry pi
UNLESS OTHERWISE SPECIFIED, DIMENSIONS ARE IN MILLIMETERS	DRAWN	ANDRES HENAO		2021-05-13	
Conception: Andres Henao carlosa.henaof@inrs.ca	CHECKED				TITLE One metre plus Top cover
Programming: Andres Henao carlosa.henaof@inrs.ca David Maignan davidmaignan@gmail.com	APPROVED				
Supervisor: Philippe Apparicio philippe.apparicio@inrs.ca	MATERIAL		FINISH		SIZE / DWG NO. / REV.
					SCALE 1:1 / WEIGHT / SHEET 3 of 10

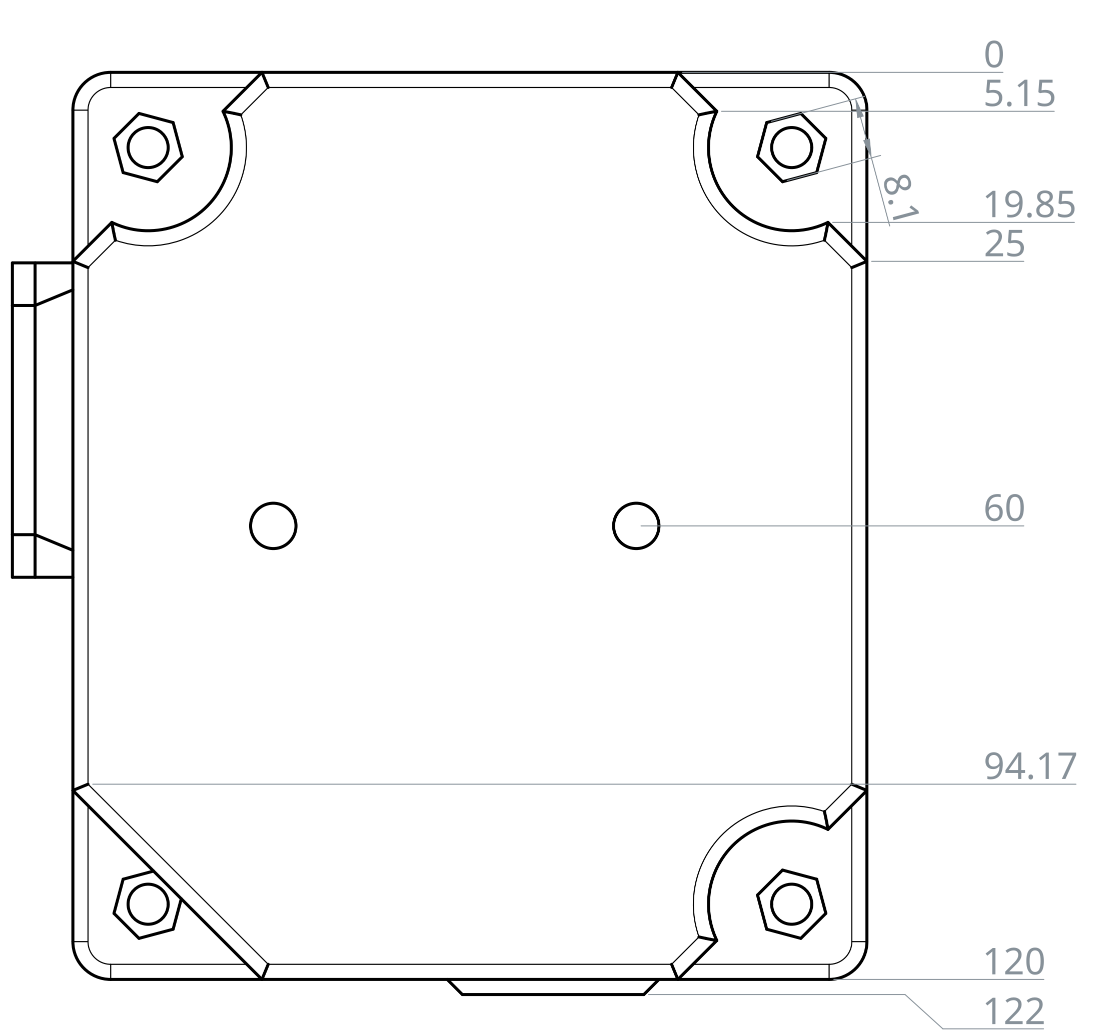

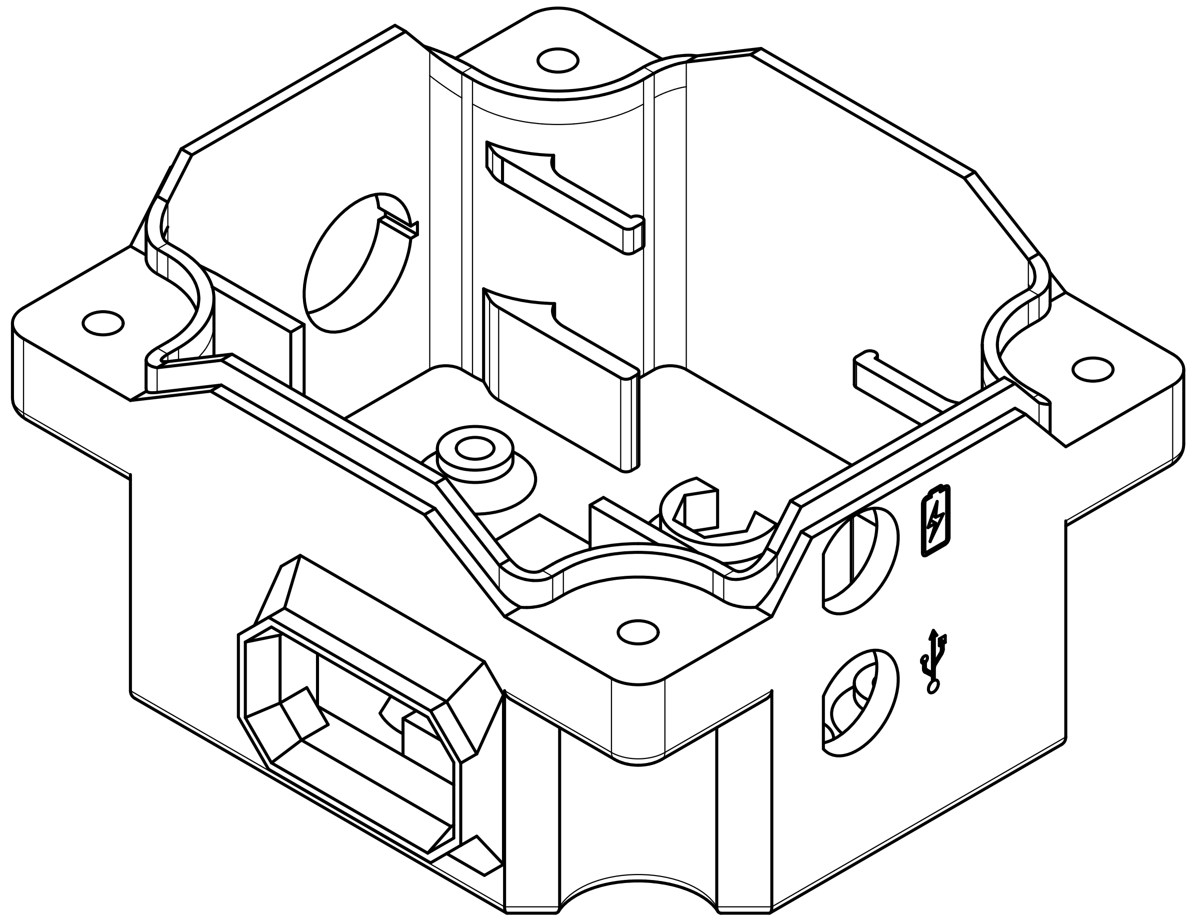

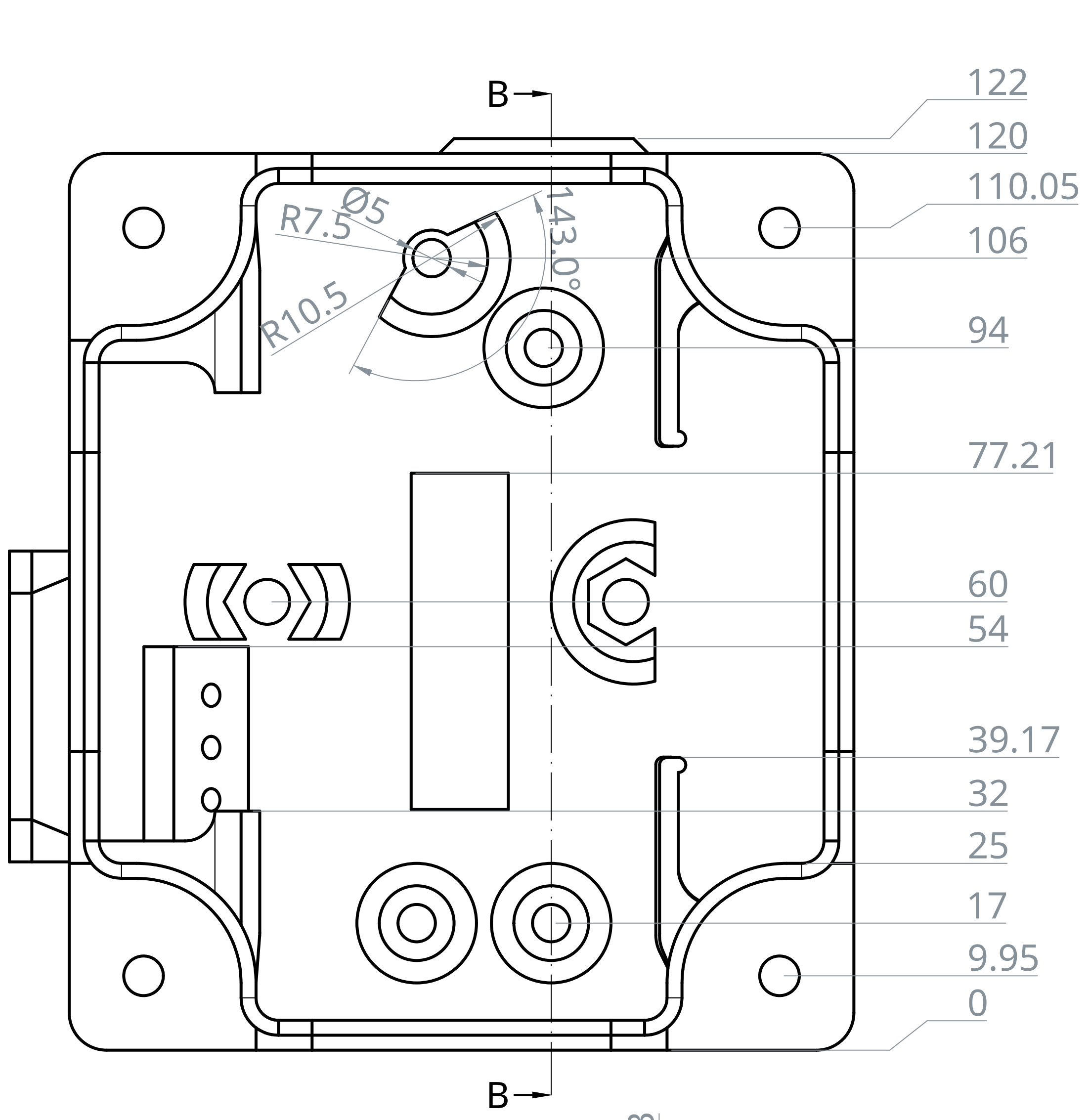

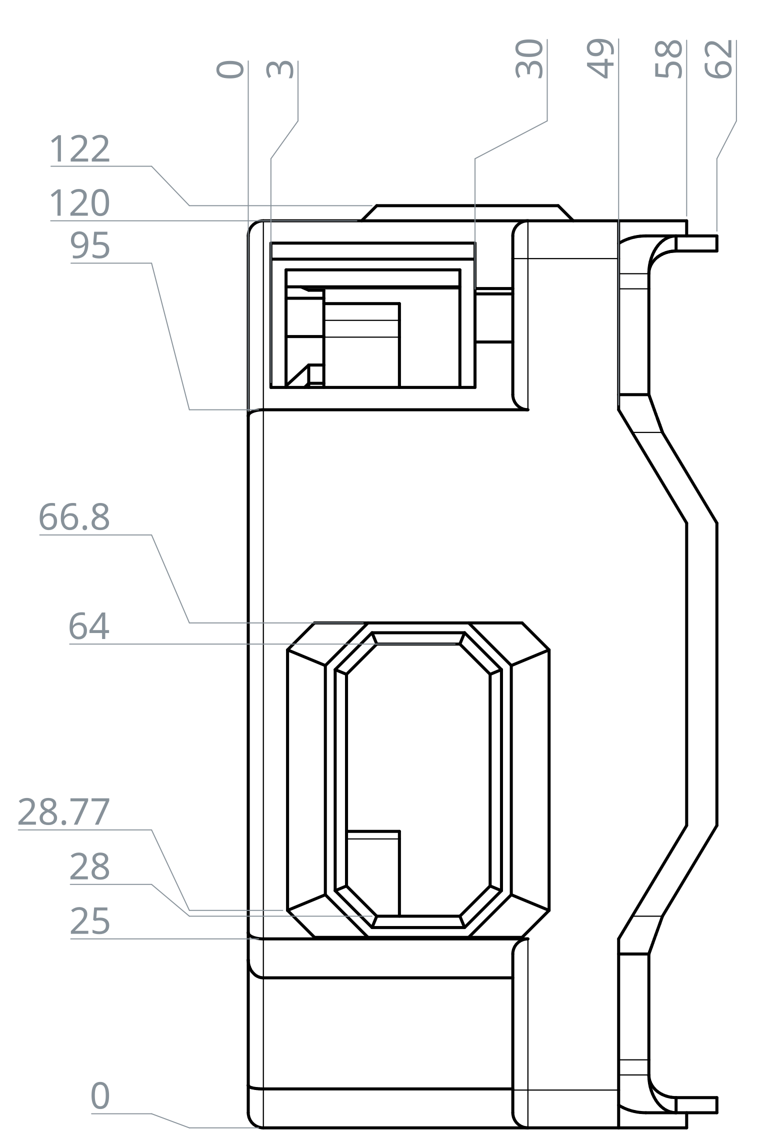

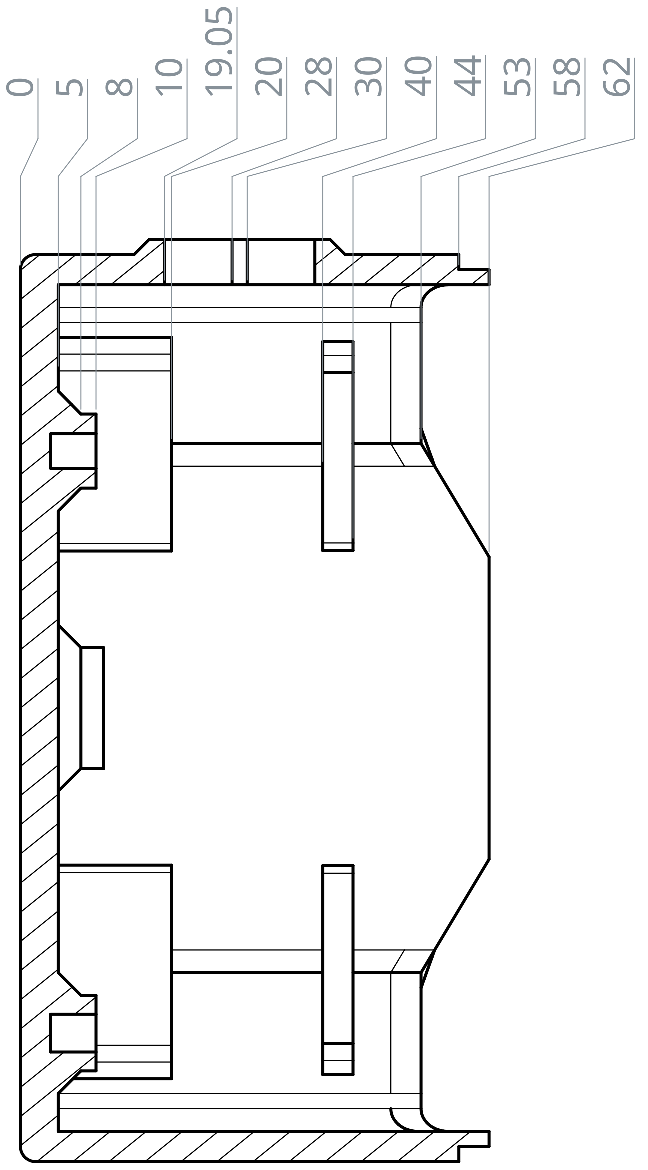

B - B

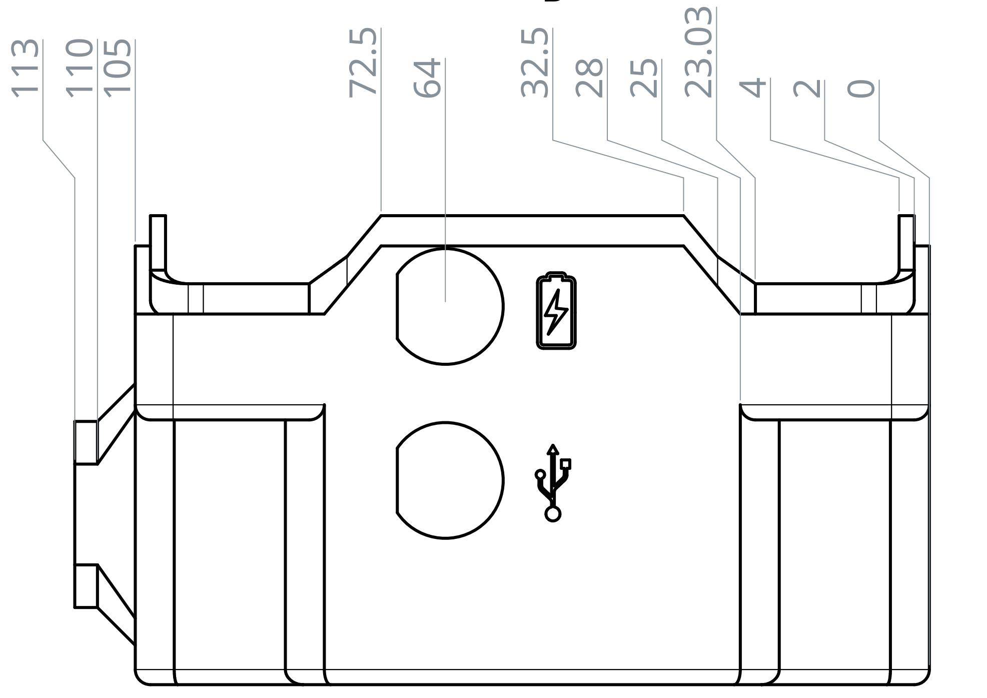

UNLESS OTHERWISE SPECIFIED, DIMENSIONS ARE IN MILLIMETERS		NAME	SIGNATURE	DATE
Conception: Andres Henao carlosa.henaof@inrs.ca	DRAWN	ANDRES HENAO		2021-05-13
Programming: Andres Henao carlosa.henaof@inrs.ca David Maignan davidmaignan@gmail.com	CHECKED			
	APPROVED			
Supervisor: Philippe Apparicio philippe.apparicio@inrs.ca	MATERIAL		FINISH	

One metre plus (1M+): a multifunctional open source sensor for bicycles based on raspberry pi

TITLE
One metre plus
Bottom Case

SIZE	DWG NO.		REV.
SCALE 1:1	WEIGHT	SHEET 4 of 10	

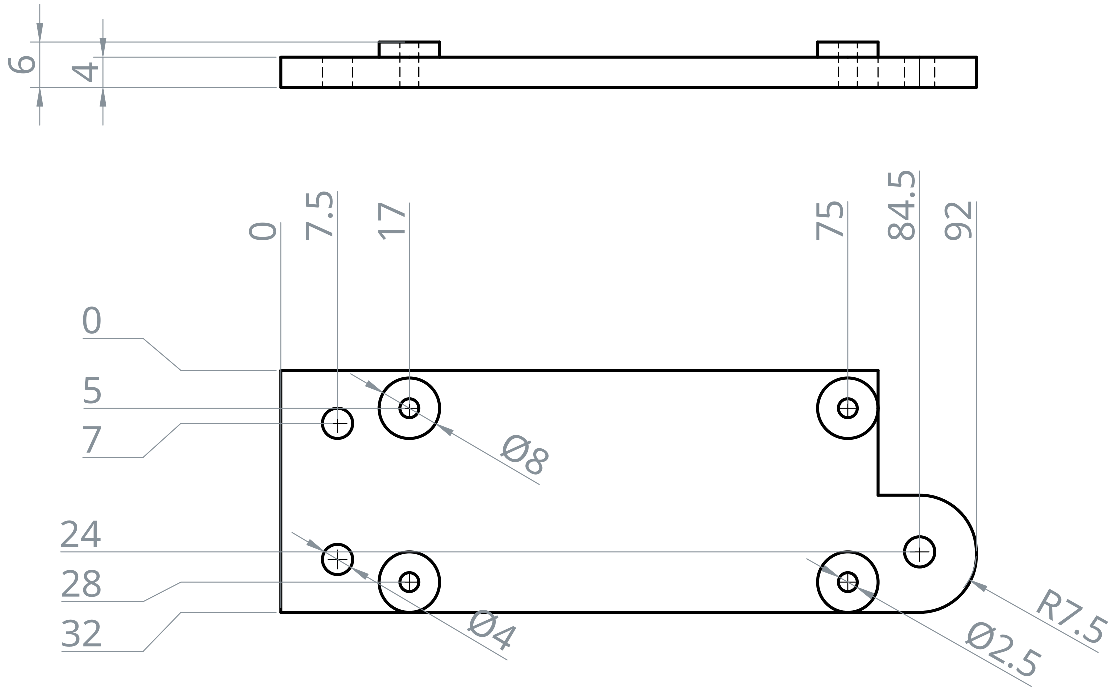

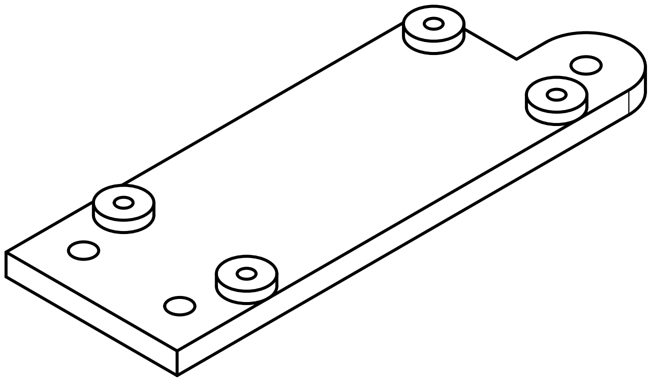

UNLESS OTHERWISE SPECIFIED, DIMENSIONS ARE IN MILLIMETERS		NAME	SIGNATURE	DATE
Conception: Andres Henao carlosa.henaof@inrs.ca	DRAWN	ANDRES HENAO		2021-05-13
Programming: Andres Henao carlosa.henaof@inrs.ca David Maignan davidmaignan@gmail.com	CHECKED			
	APPROVED			
Supervisor: Philippe Apparicio philippe.apparicio@inrs.ca	MATERIAL		FINISH	

One metre plus (1M+): a multifunctional open source sensor for bicycles based on raspberry pi

TITLE: One metre plus Raspberry pi support

SIZE	DWG NO.	REV.
A4		

SCALE	WEIGHT	SHEET
1:1		5 of 10

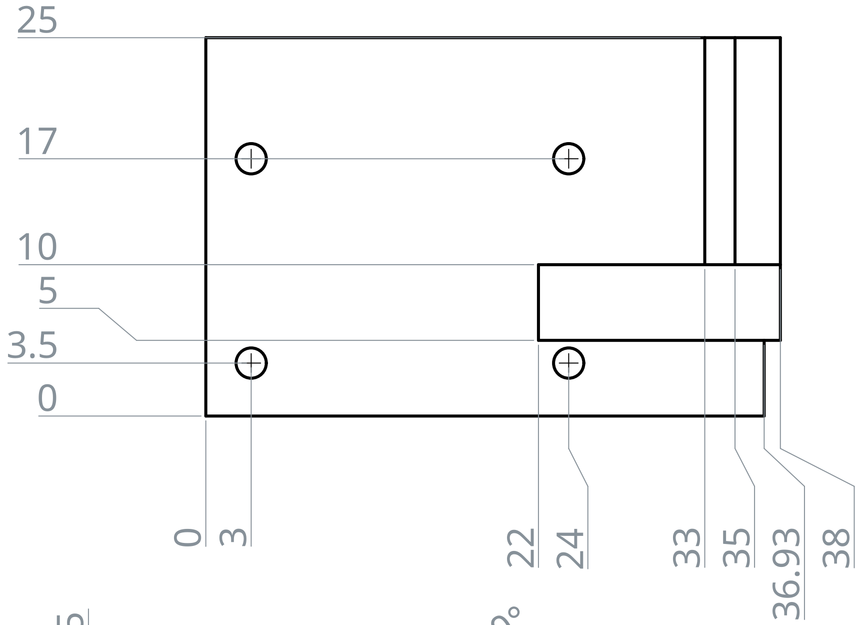

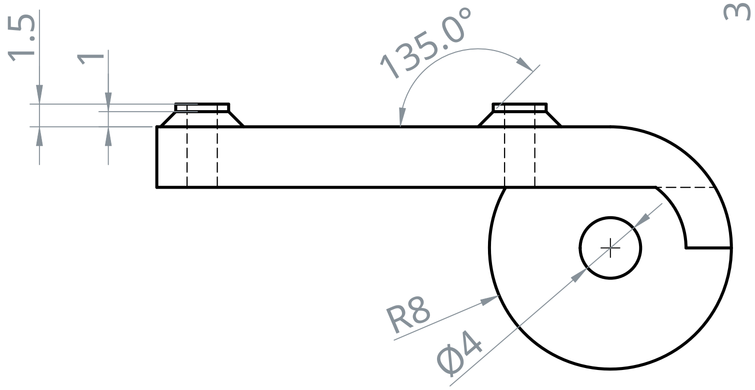

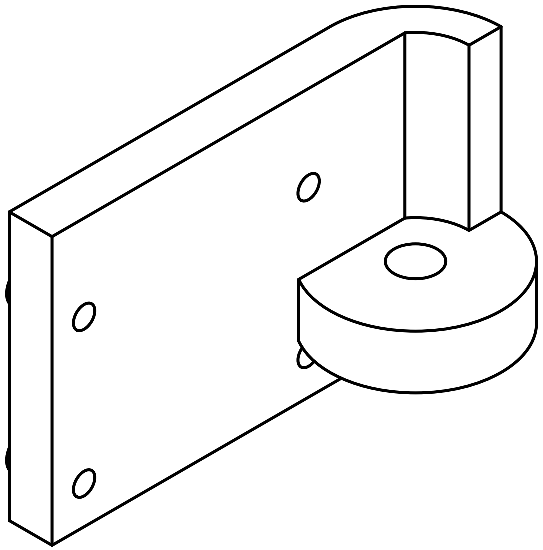

UNLESS OTHERWISE SPECIFIED, DIMENSIONS ARE IN MILLIMETERS

Conception: Andres Henao
carlosa.henaof@inrs.ca
Programming:
Andres Henao
carlosa.henaof@inrs.ca
David Maignan
davidmaignan@gmail.com
Supervisor: Philippe Apparicio
philippe.apparicio@inrs.ca

	NAME	SIGNATURE	DATE
DRAWN	ANDRES HENAO		2021-05-13
CHECKED			
APPROVED			

MATERIAL

FINISH

One metre plus (1M+): a multifunctional open source sensor for bicycles based on raspberry pi

TITLE

One metre plus Camera support

SIZE A4

DWG NO.

REV.

SCALE 2:1

WEIGHT

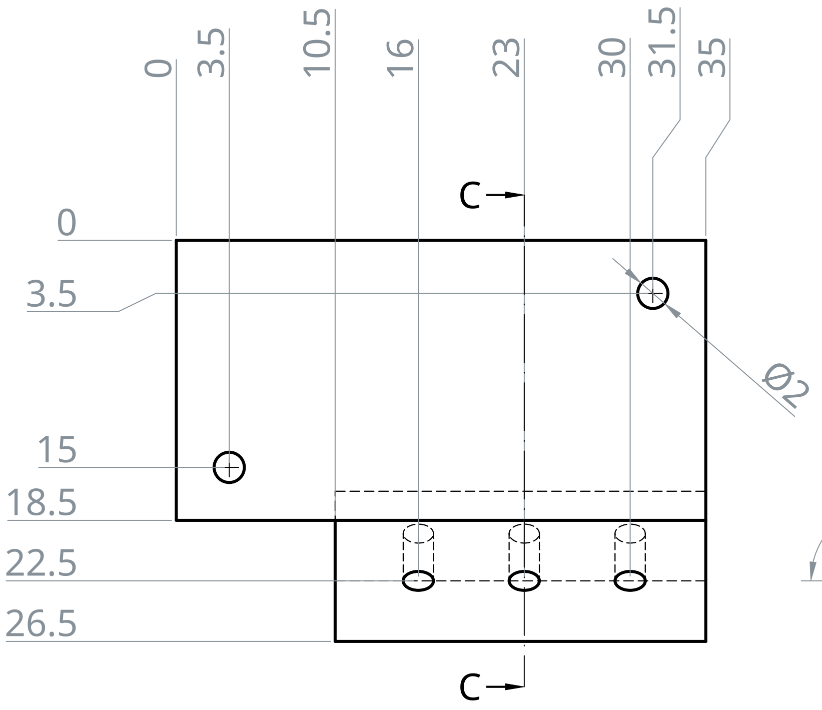

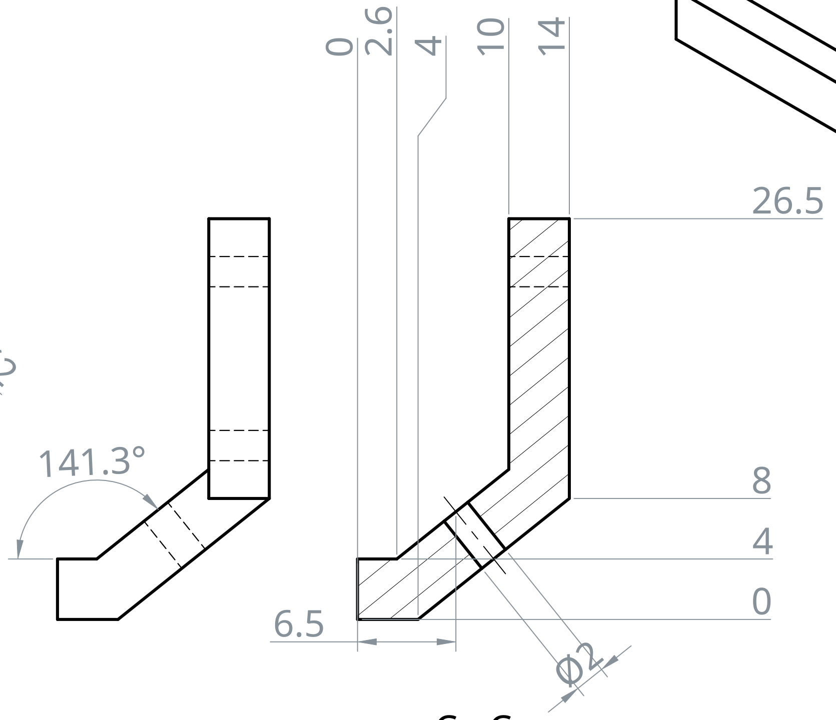

C - C

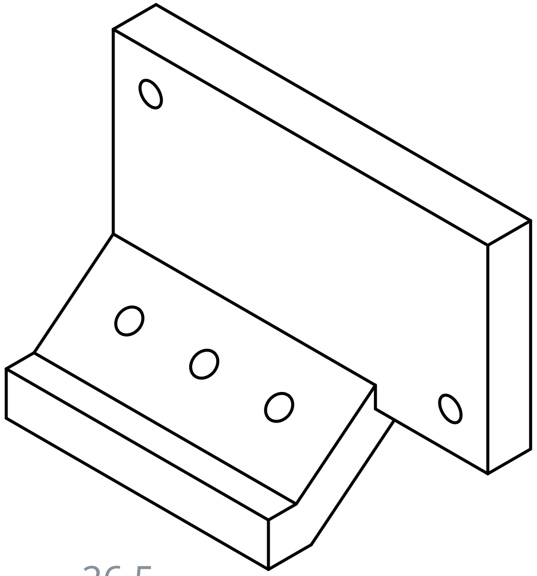

UNLESS OTHERWISE SPECIFIED, DIMENSIONS ARE IN MILLIMETERS		NAME	SIGNATURE	DATE	One metre plus (1M+): a multifunctional open source sensor for bicycles based on raspberry pi
Conception: Andres Henao carlosa.henaof@inrs.ca	DRAWN	ANDRES HENAO		2021-05-13	
Programming: Andres Henao carlosa.henaof@inrs.ca David Maignan davidmaignan@gmail.com	CHECKED				TITLE One metre plus Distance sensor support
	APPROVED				
Supervisor: Philippe Apparicio philippe.apparicio@inrs.ca	MATERIAL		FINISH		SIZE A4 / DWG NO. / REV.
					SCALE 2:1 / WEIGHT / SHEET 7 of 10

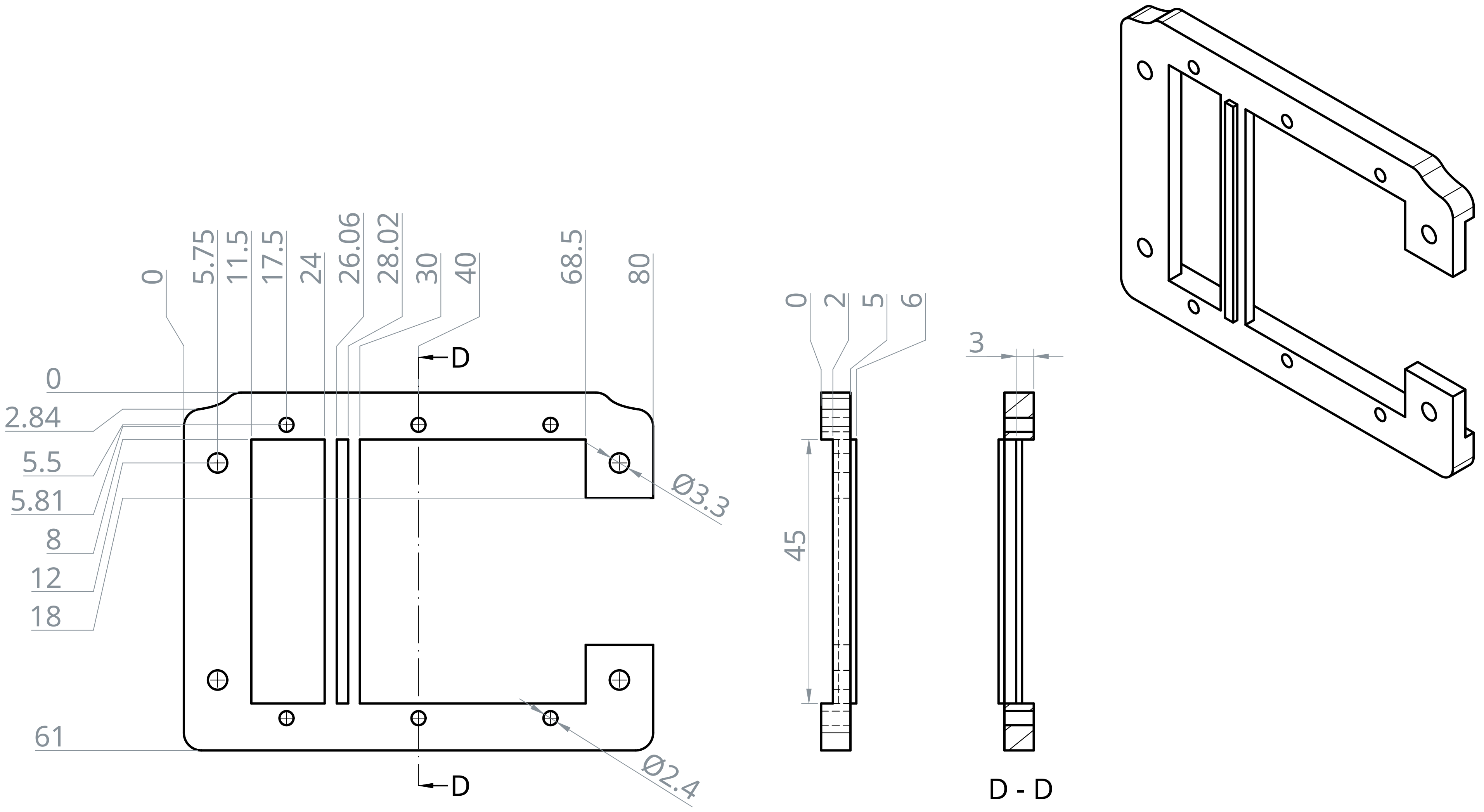

UNLESS OTHERWISE SPECIFIED, DIMENSIONS ARE IN MILLIMETERS		NAME	SIGNATURE	DATE
Conception: Andres Henao carlosa.henaof@inrs.ca	DRAWN	ANDRES HENAO		2021-05-13
Programming: Andres Henao carlosa.henaof@inrs.ca David Maignan davidmaignan@gmail.com	CHECKED			
	APPROVED			
Supervisor: Philippe Apparicio philippe.apparicio@inrs.ca	MATERIAL		FINISH	

One metre plus (1M+): a multifunctional open source sensor for bicycles based on raspberry pi

TITLE

One metre plus
Touch screen support

SIZE	DWG NO.	REV.
A4		

SCALE	WEIGHT	SHEET
1:1		8 of 10

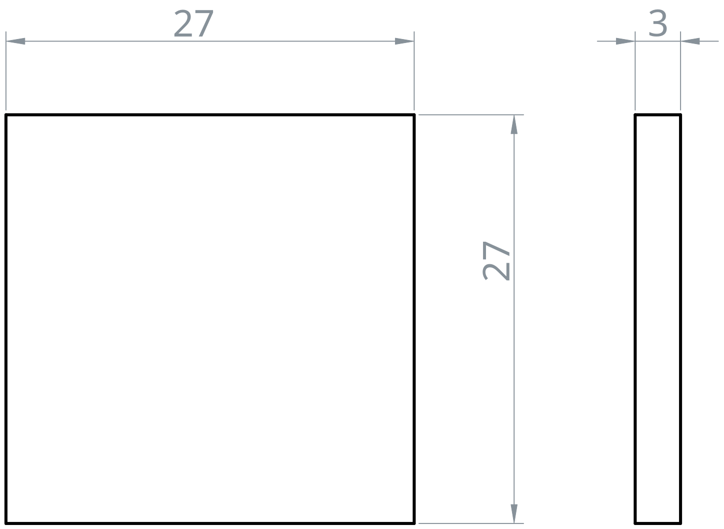

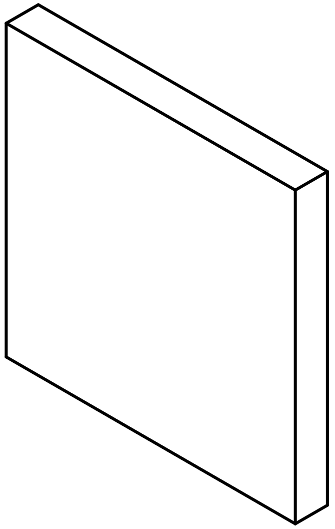

UNLESS OTHERWISE SPECIFIED, DIMENSIONS ARE IN MILLIMETERS		NAME	SIGNATURE	DATE	One metre plus (1M+): a multifunctional open source sensor for bicycles based on raspberry pi
Conception: Andres Henao carlosa.henaof@inrs.ca	DRAWN	ANDRES HENAO		2021-05-13	
Programming: Andres Henao carlosa.henaof@inrs.ca David Maignan davidmaignan@gmail.com	CHECKED				TITLE One metre plus Acrylic cover
	APPROVED				
Supervisor: Philippe Apparicio philippe.apparicio@inrs.ca	MATERIAL		FINISH		SIZE A4 / DWG NO. / REV.
					SCALE 2:1 / WEIGHT / SHEET 9 of 10

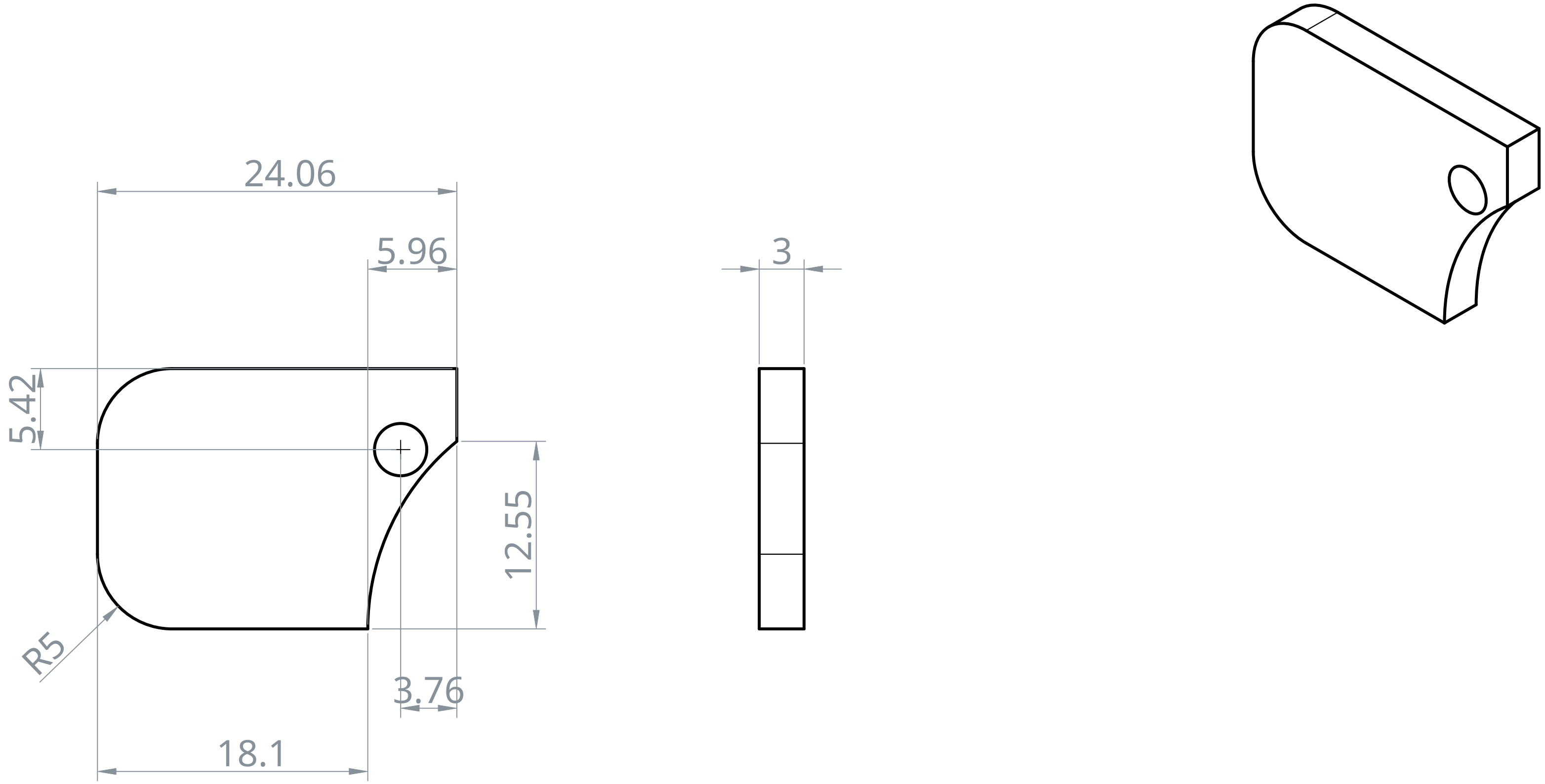

UNLESS OTHERWISE SPECIFIED, DIMENSIONS ARE IN MILLIMETERS		NAME	SIGNATURE	DATE	One metre plus (1M+): a multifunctional open source sensor for bicycles based on raspberry pi
Conception: Andres Henao carlosa.henaof@inrs.ca	DRAWN	ANDRES HENAO		2021-05-13	
Programming: Andres Henao carlosa.henaof@inrs.ca David Maignan davidmaignan@gmail.com	CHECKED				TITLE One metre plus Acrylic cover
	APPROVED				
Supervisor: Philippe Apparicio philippe.apparicio@inrs.ca	MATERIAL	FINISH			SIZE A4 · DWG NO. · REV.
					SCALE 2:1 · WEIGHT · SHEET 10 of 10

Supplementary material for the article: One meter plus (1M+), a multifunctional open source sensor for bicycles based on raspberry pi.

3. Connection diagram

1M+ Sensor

One meter plus (1M+)
A multifunctional open source sensor for bicycles based on raspberry pi.
Connection diagram

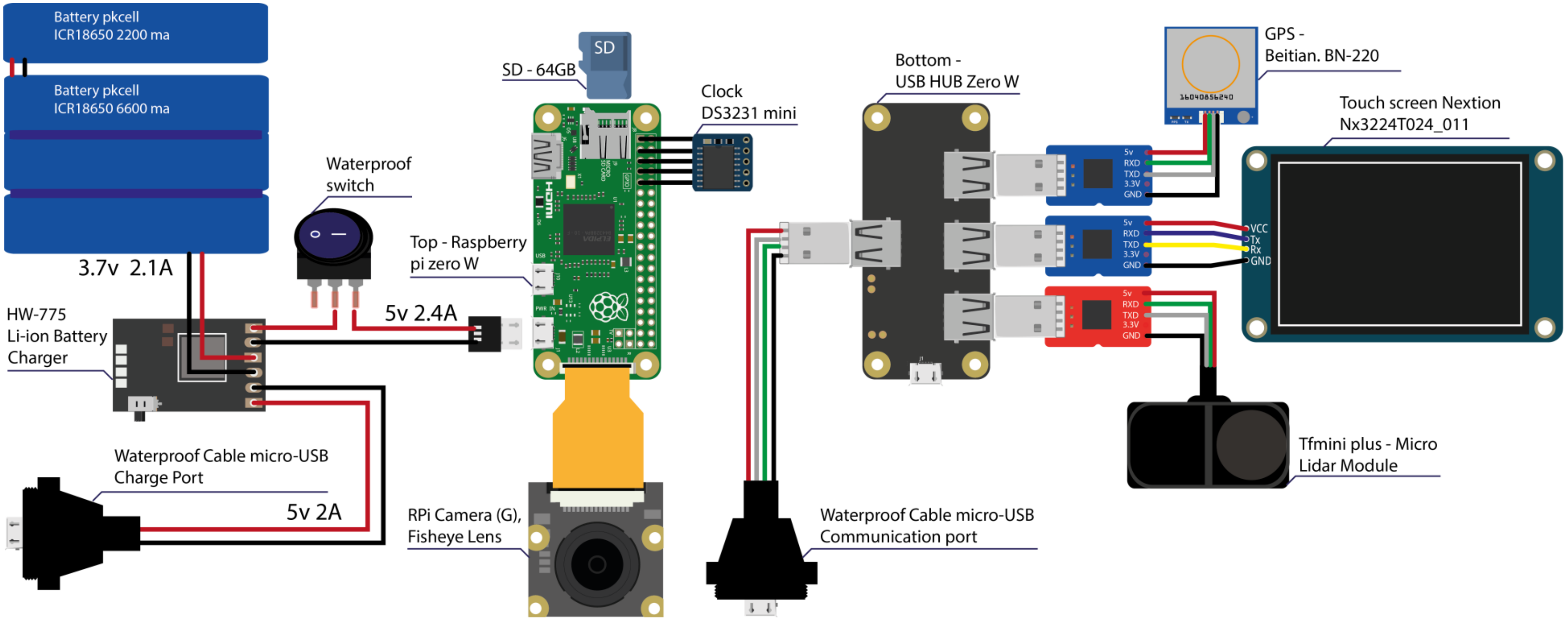

Schematic connection diagram

Supplementary material for the article: One meter plus (1M+), a multifunctional open source sensor for bicycles based on raspberry pi.

4. Guide to counting overtaking manoeuvres

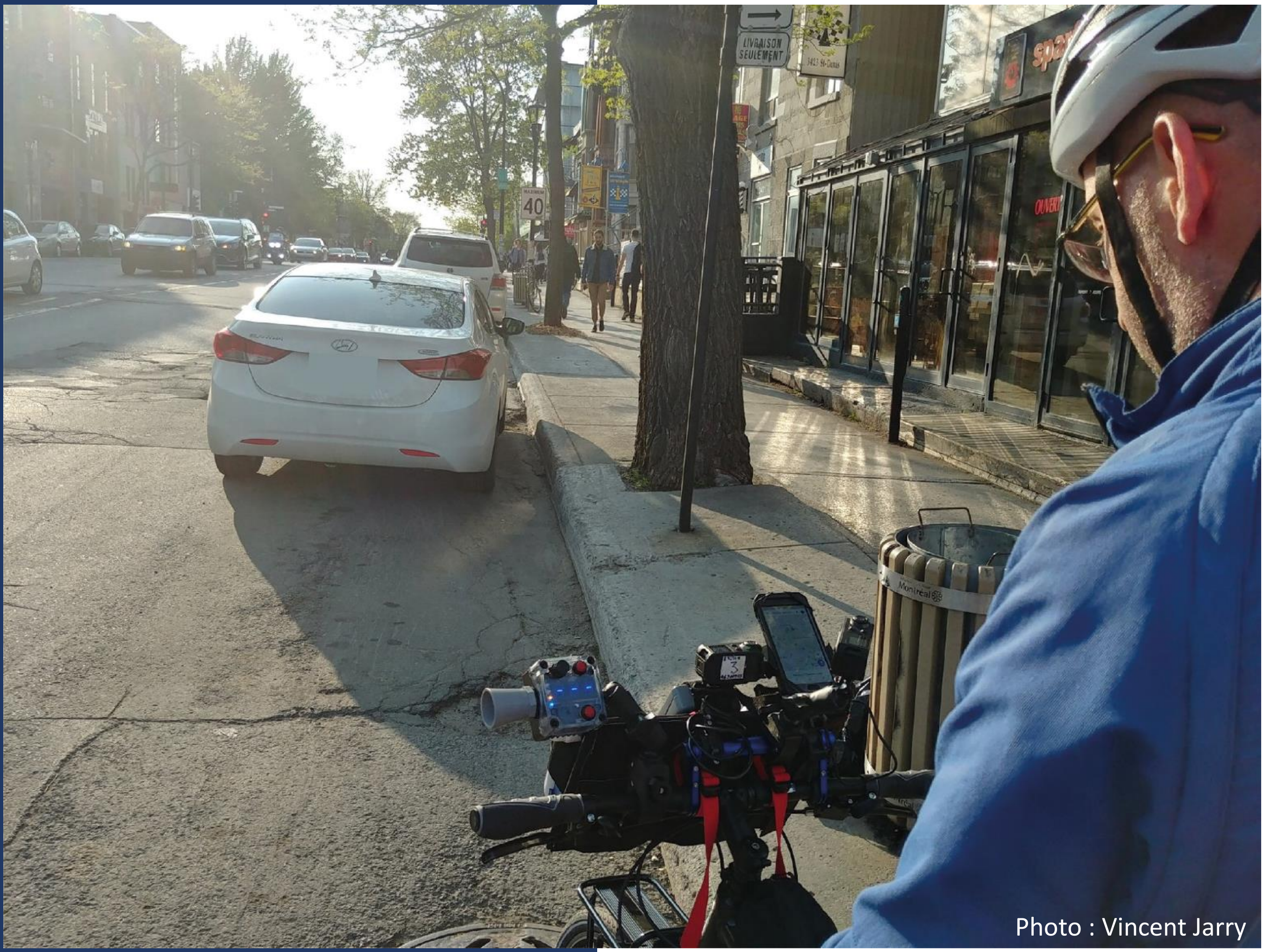

Photo : Vincent Jarry

Institut national de la recherche scientifique

Guide to Counting Overtaking Manoeuvres

Supplementary Material to the Article: One metre plus (1M+): a multifunctional open source sensor for bicycles based on raspberry pi.

Objective: Identify the characteristics of overtaking manoeuvres over cyclists in Montreal.

Andrés Henao Florez,

Master's student in Urban Studies (INRS)
Member of the Environmental Equity Laboratory (LAEQ)
carlosa.henaof@inrs.ca

Philippe Apparicio,

Full Professor at INRS
Director of the Environmental Equity Laboratory (LAEQ)
philippe.apparicio@inrs.ca

1. Type of Overtaking Manoeuvres

In this section, the different types of overtaking manoeuvres considered in the research project are described.

1.1. Type 1: Passing alongside and returning to the cyclist's line.

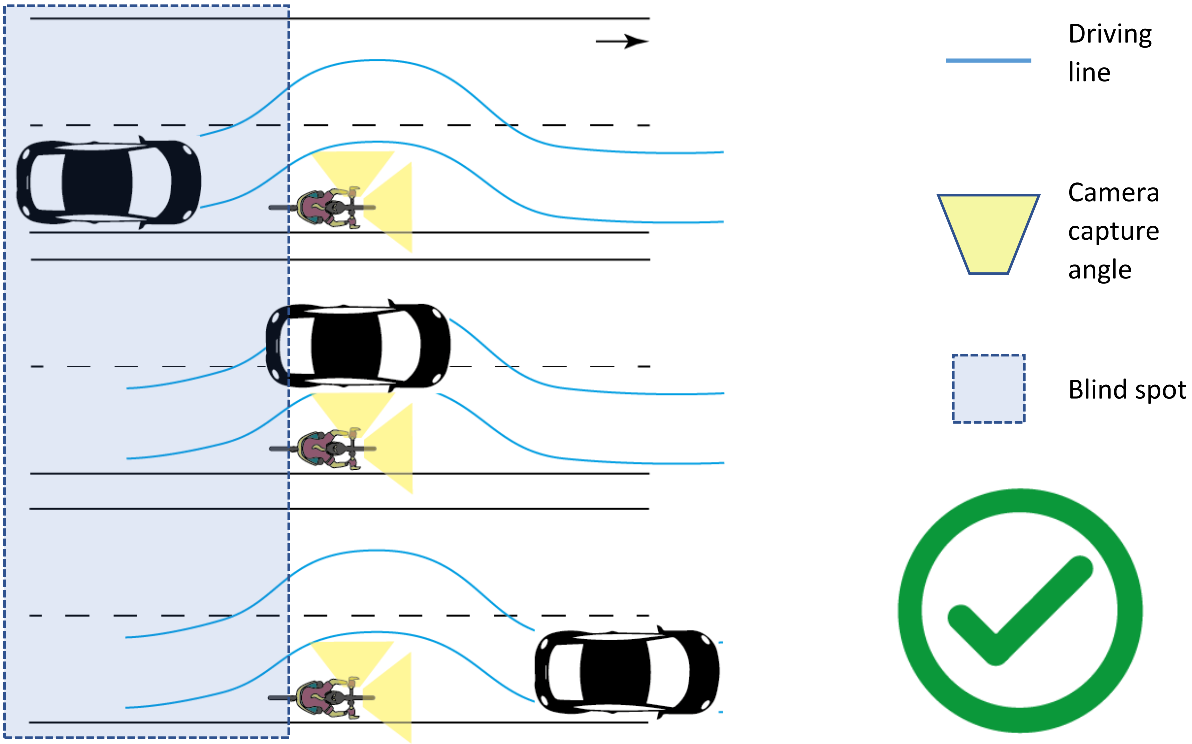

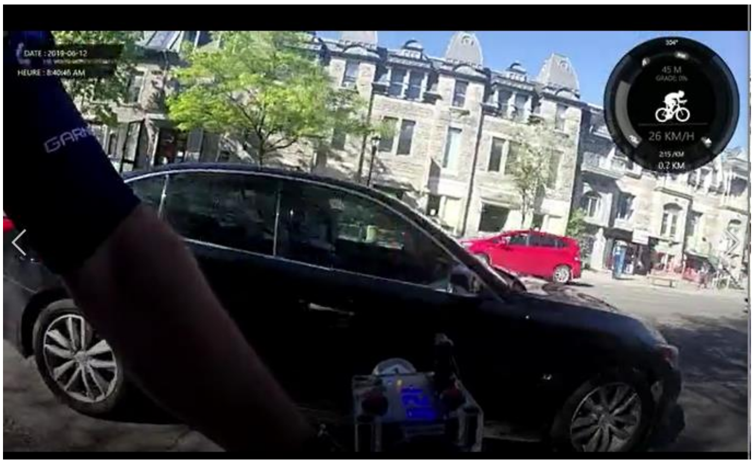

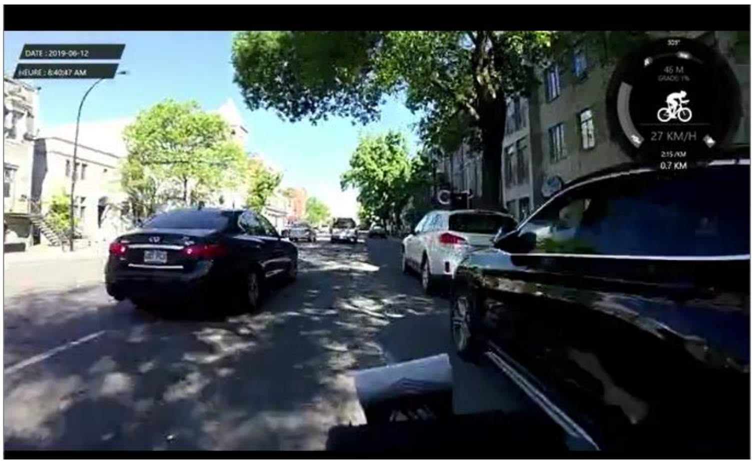

1.2. Type 2: Passing alongside and returning to the cyclist's line.

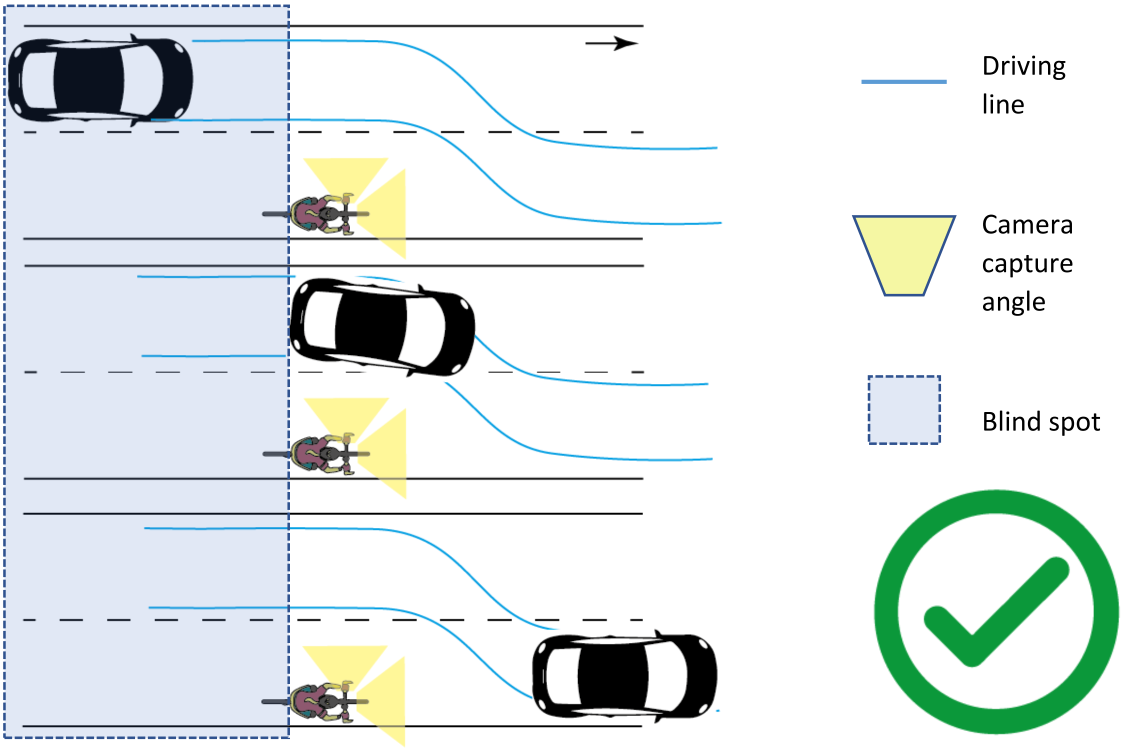

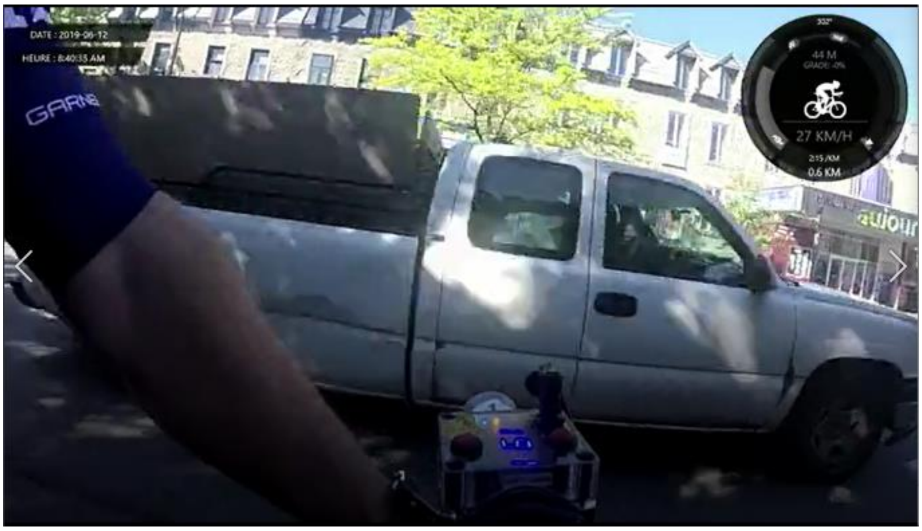

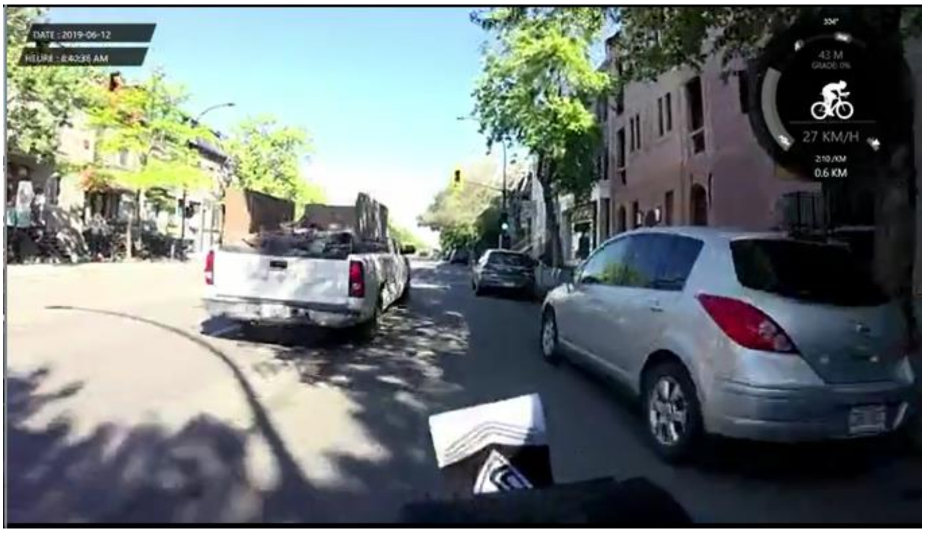

1.3. Type 3: Passing alongside a cycle lane.

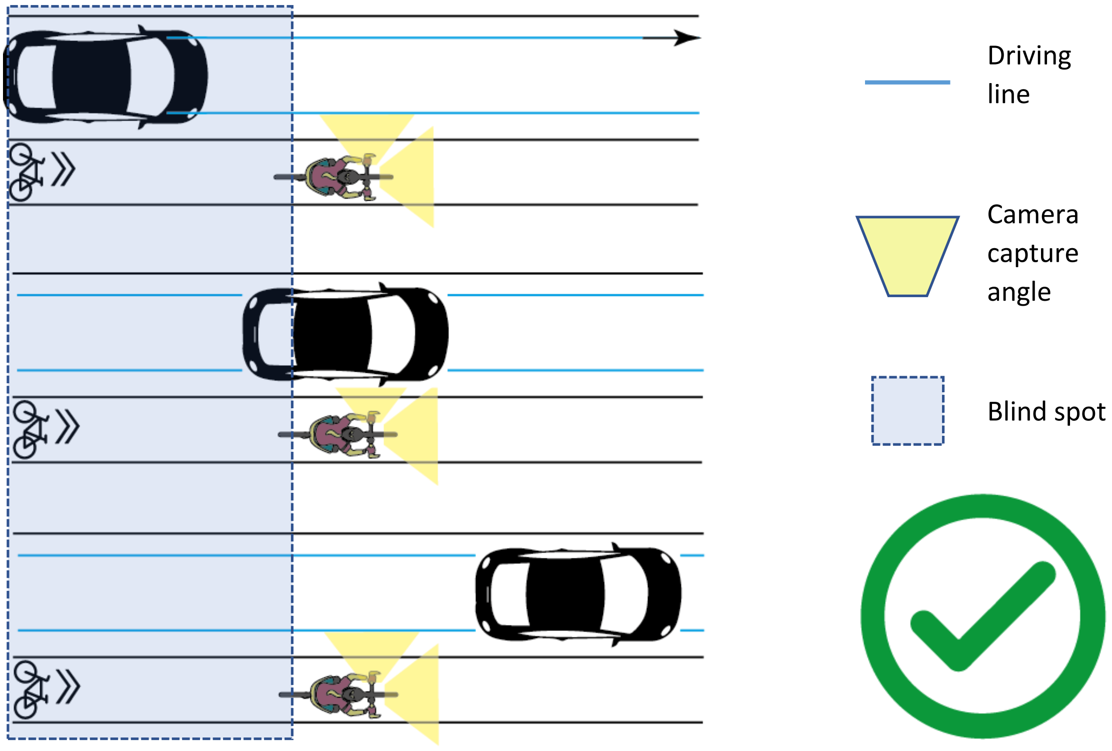

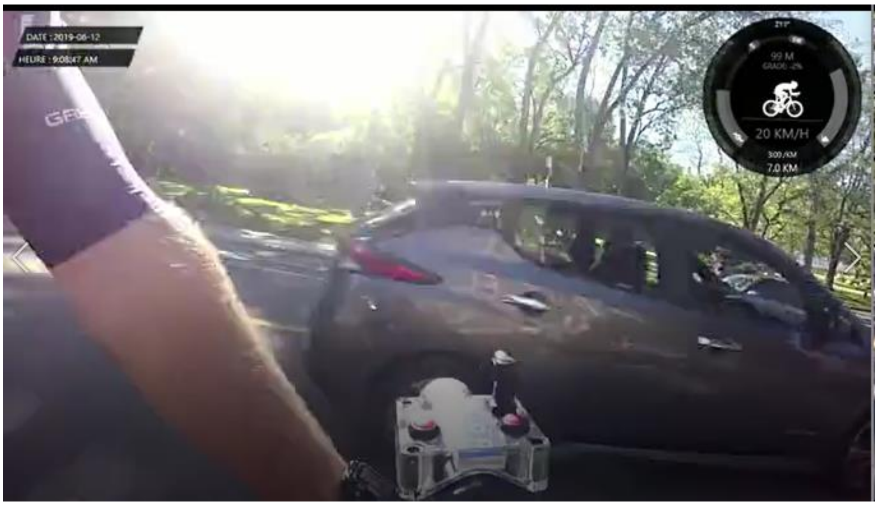

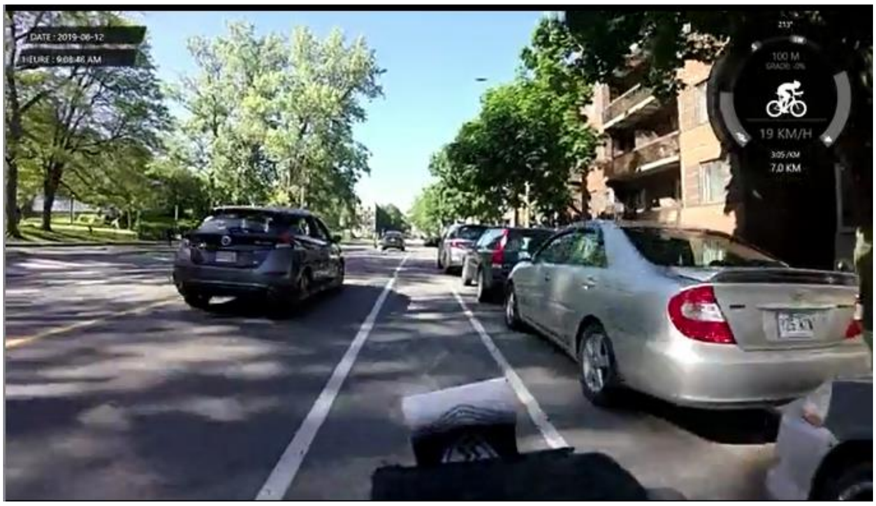

1.4. Type 4: Overtaking a cyclist by encroaching on their lane. The dividing line must be visible.

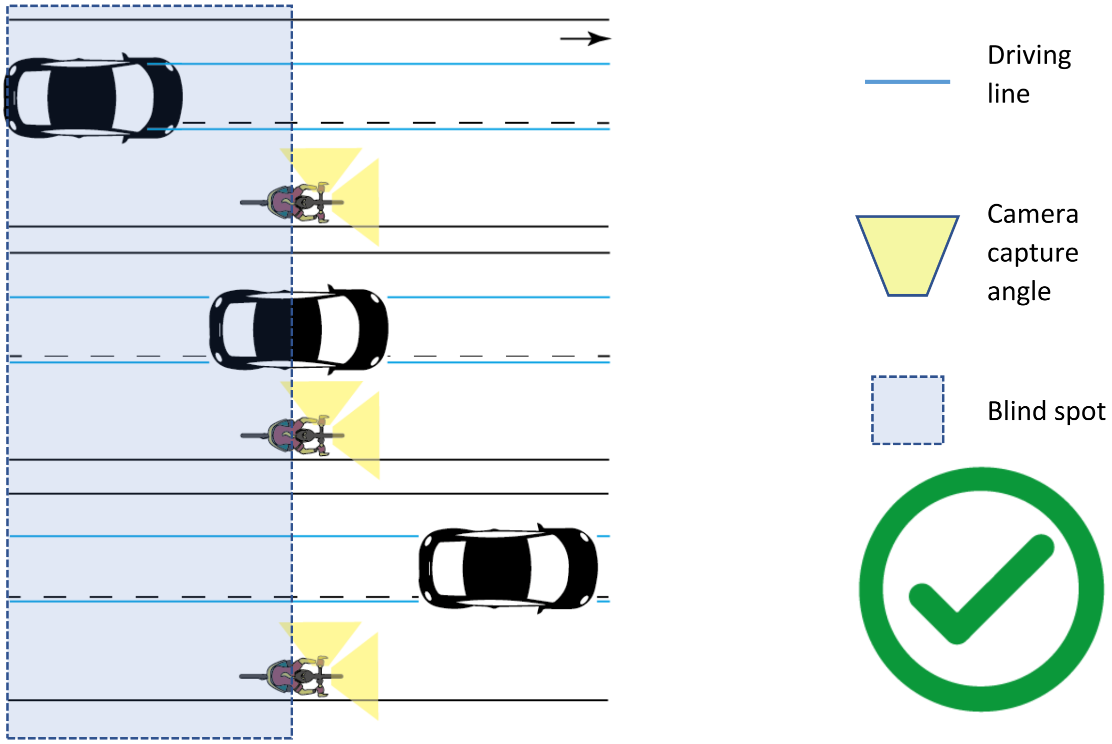

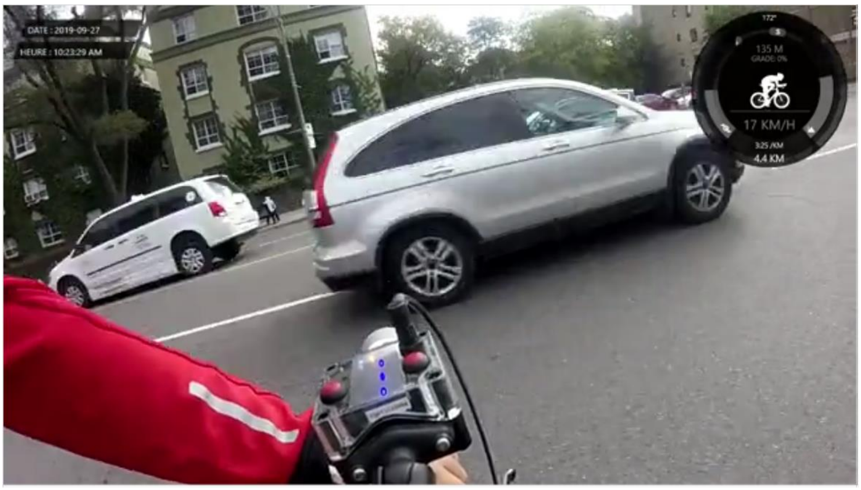

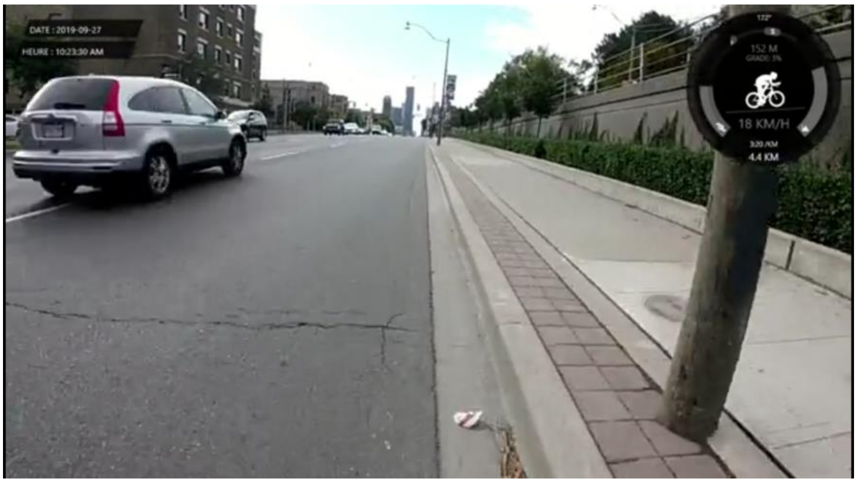

1.5. Passing a cyclist in the second lane is not considered an overtaking manoeuver. The dividing line must be visible.

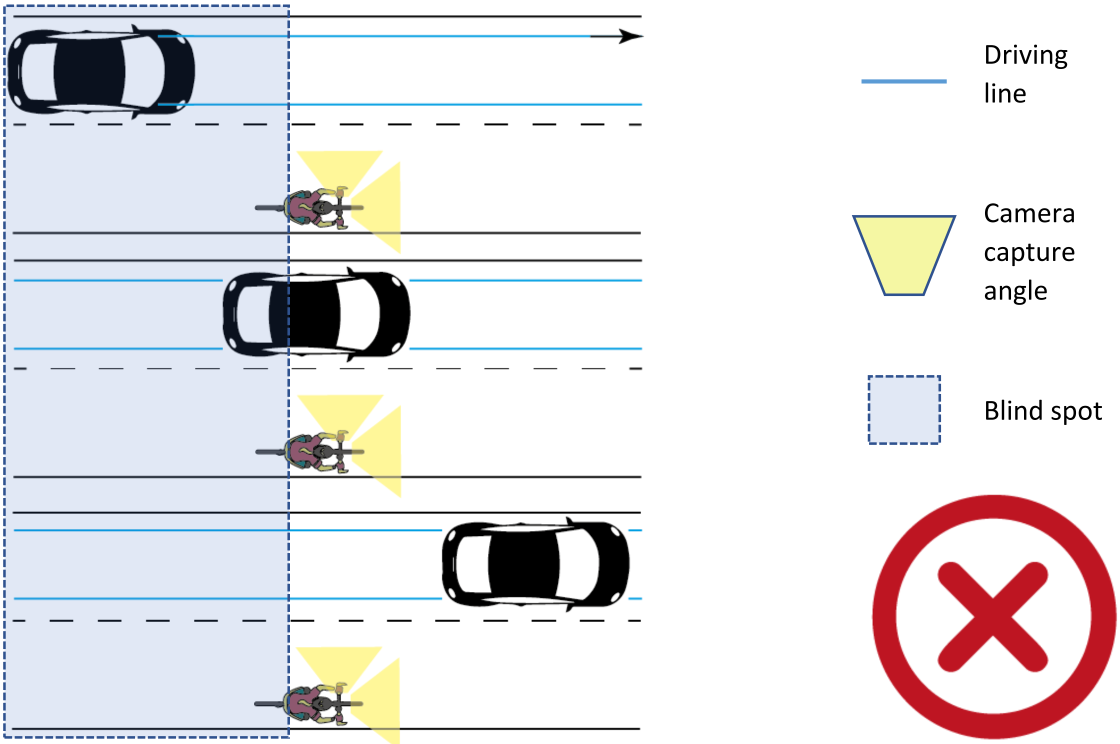

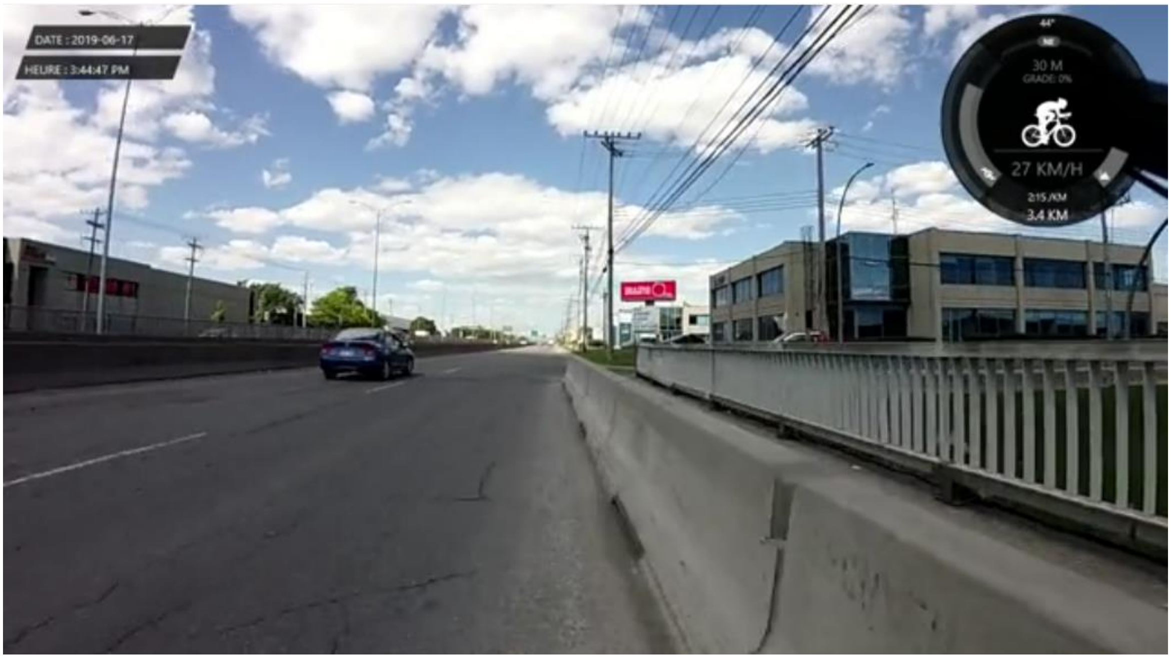

1.6. Type 5: In the absence of a demarcation line or a blurred demarcation, any passage is considered as an overtaking manoeuver.

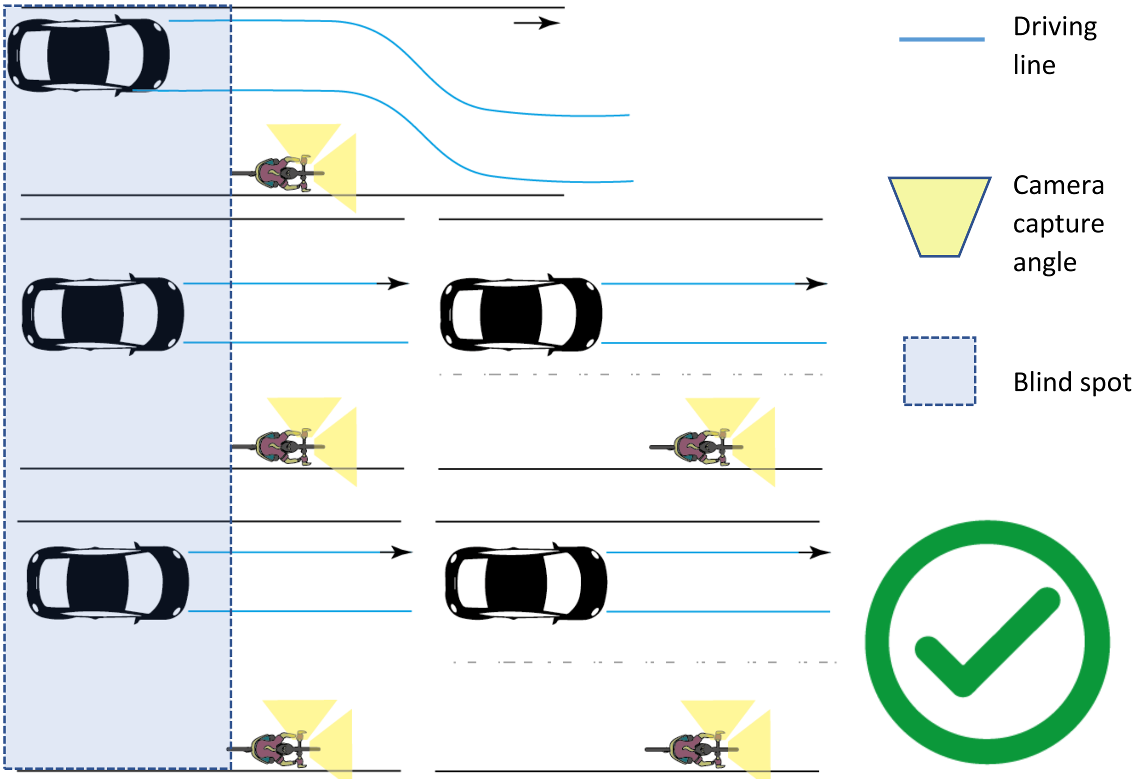

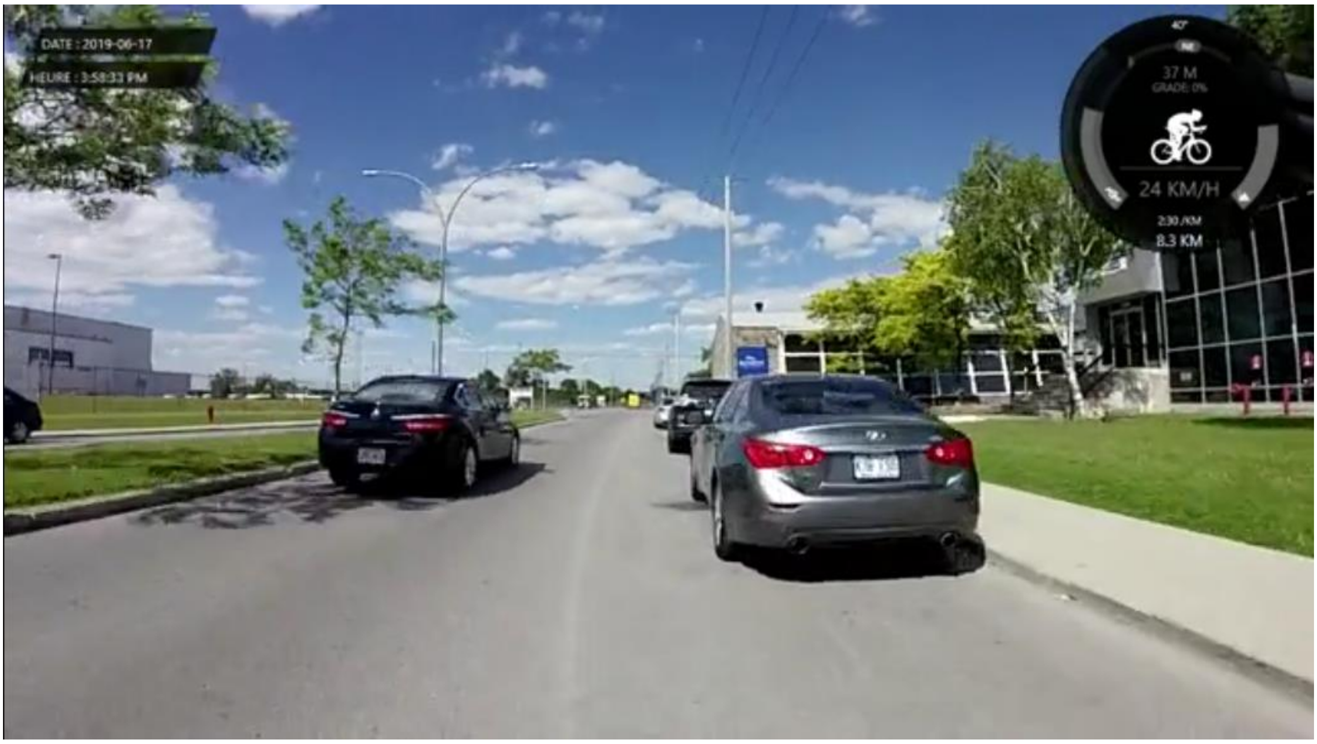

1.1. Type 6: When vehicles are parked on the road and a vehicle approaches a cyclist from the second or third lane: the examples in the figure should be considered as an overtaking manoeuver.

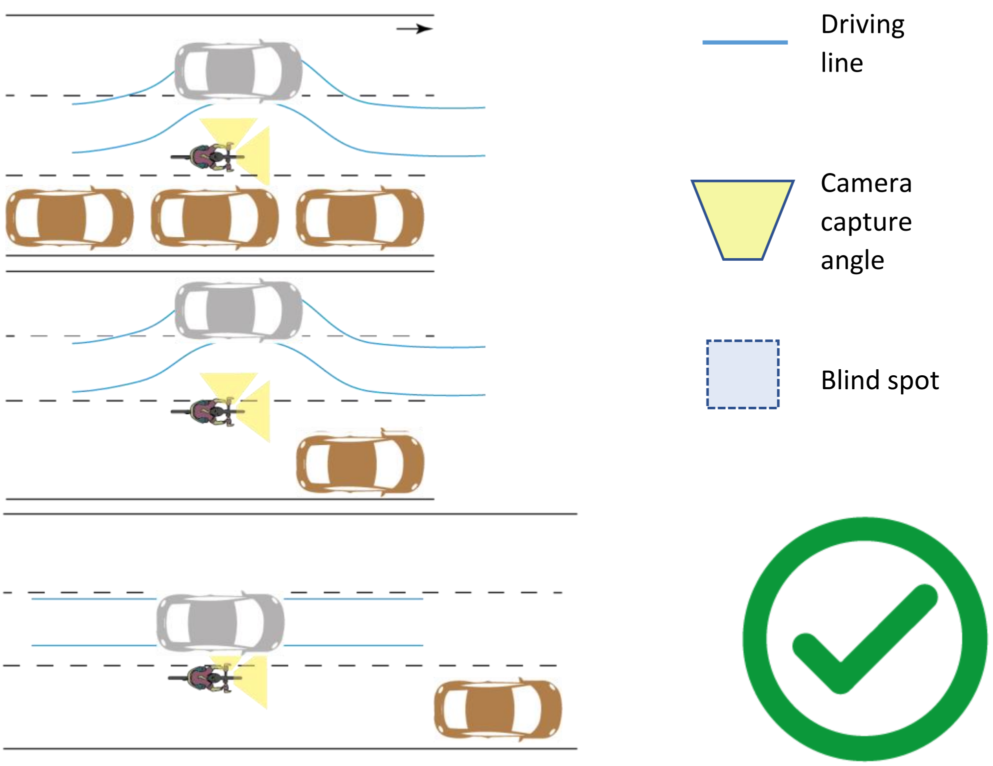

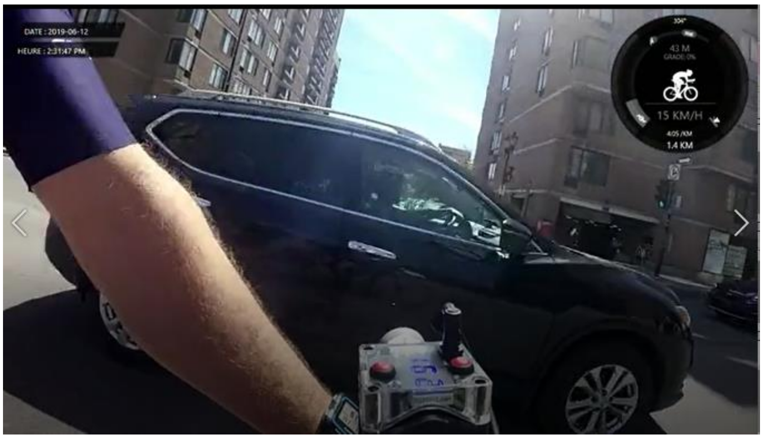

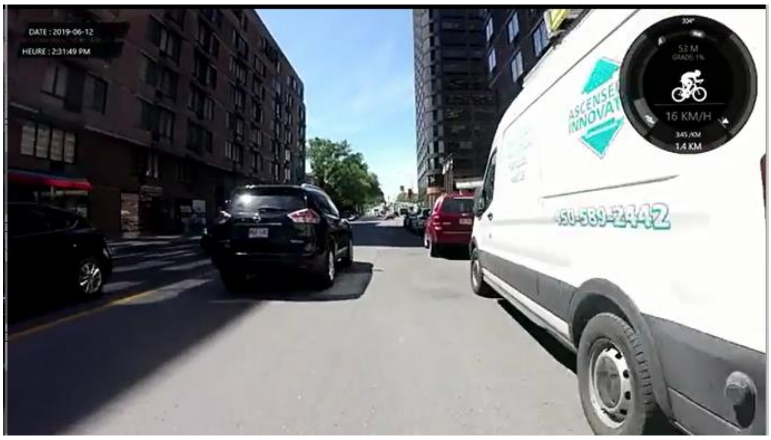

1.2. Overtaking during a red light: the passage of the parked car (the black vehicle in the figure) that starts to move alongside the cyclist is not considered as an overtaking manoeuver. However, the passing of the second car (blue) is considered as overtaking, on the condition that the last car is outside the angle of vision of the side camera.

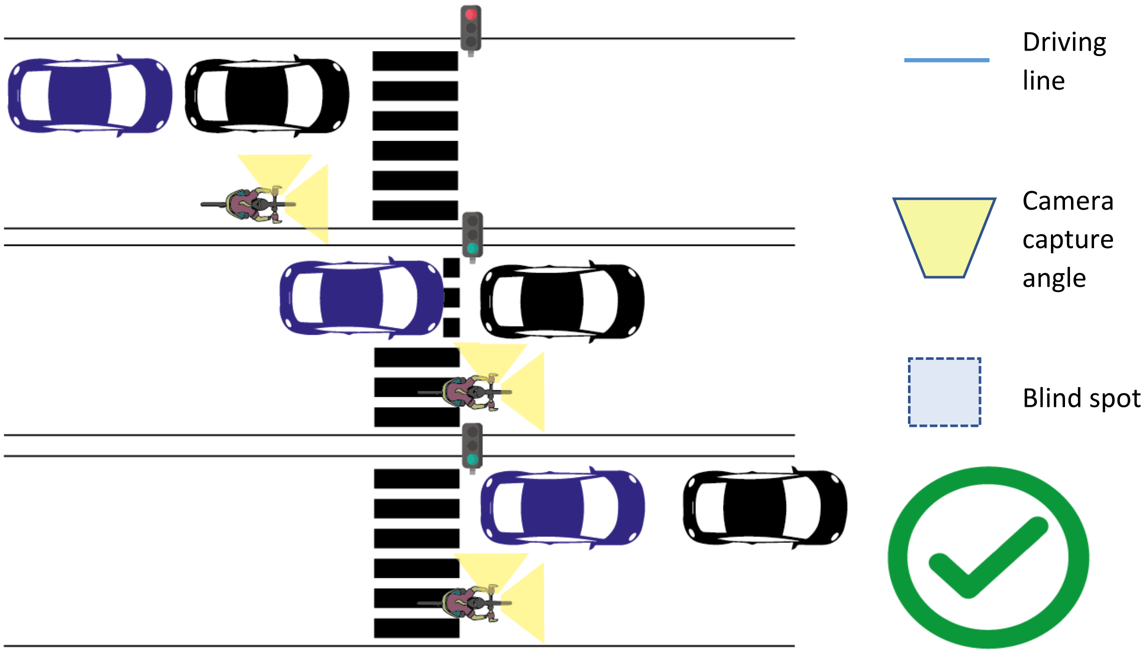

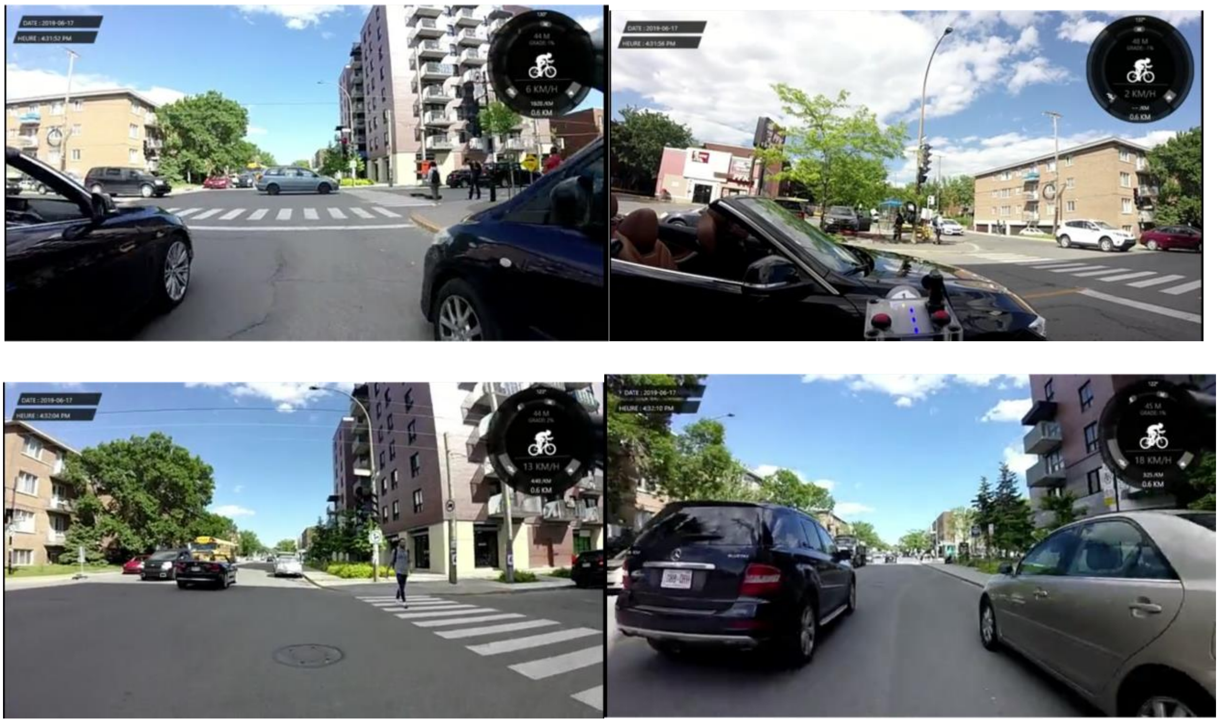

1.1. Overtaking during an intersection: If the cyclist is completely stopped at a stop-controlled intersection, the traffic light rule applies. A stopped car (in the figure, the black car), which then starts to move next to the cyclist, is not considered to be an overtaking manoeuver. The passing of the second (blue) car is considered to be overtaking, on the condition that the last car is outside the angle of vision of the side camera.

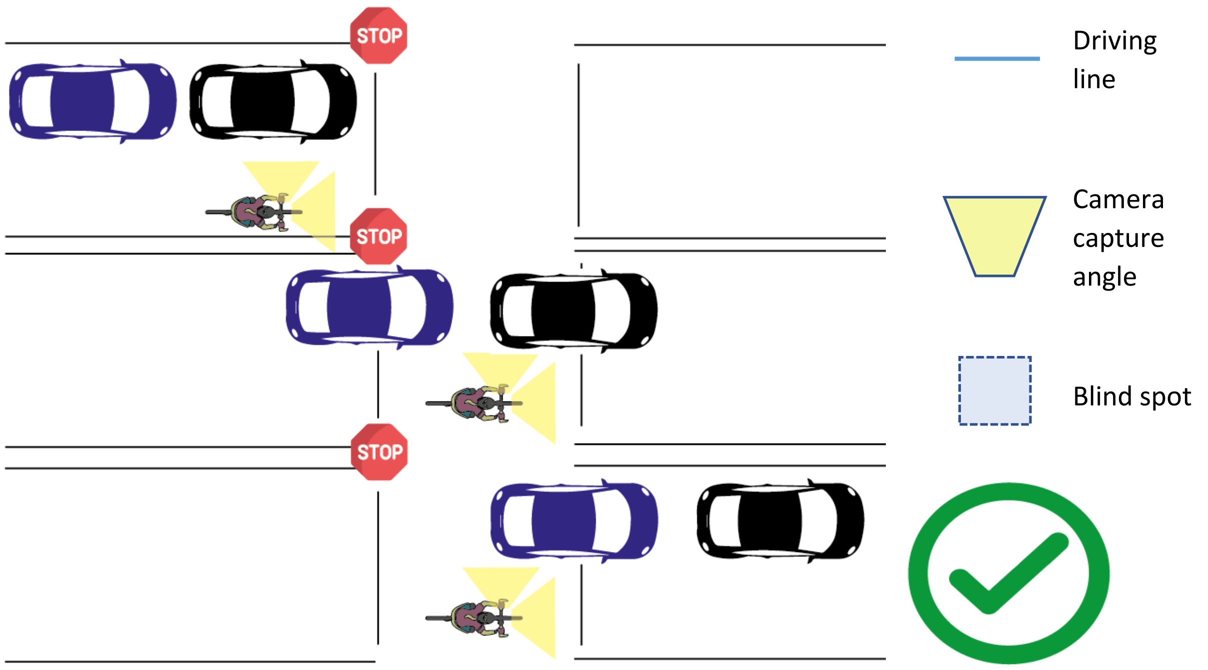

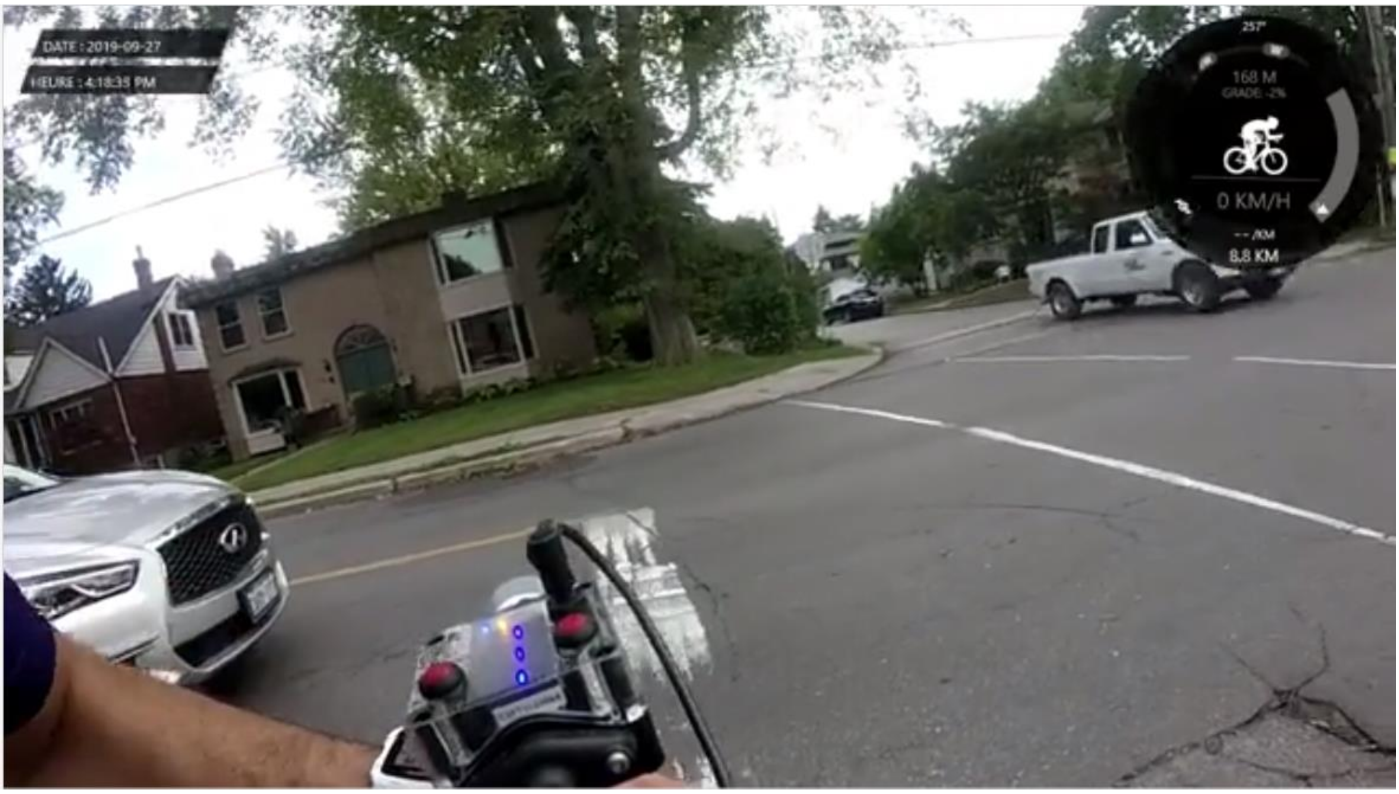

1.2. Other Considerations

If the cyclist is riding in a double cycle lane or if the cyclist is surrounded by vehicles parked in the road, the passing of the vehicle is not considered an overtaking manoeuver.

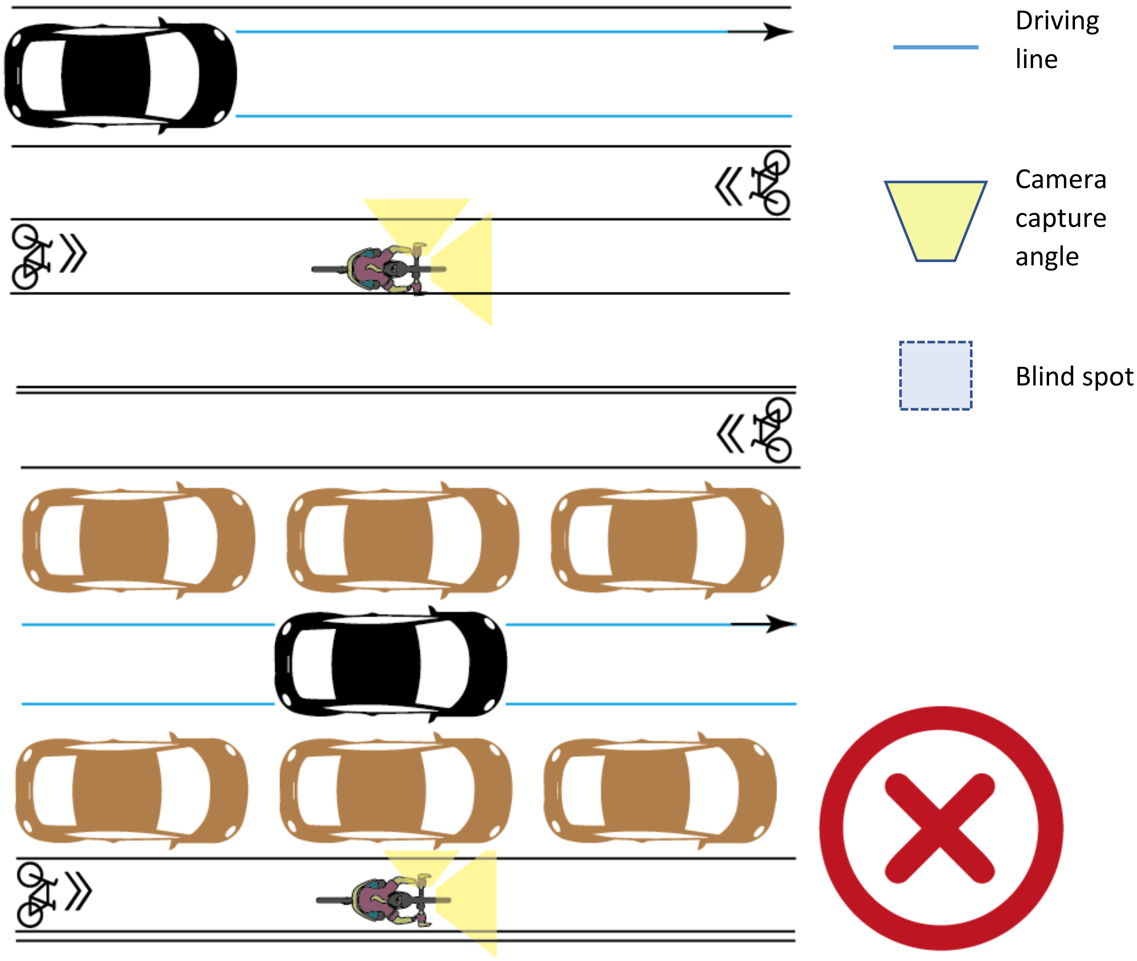

2. Type of Vehicle:

This section defines the five different types of vehicles in the research:

1. Standard car
2. Pick-up
3. Truck
4. Bus
5. Other

2.1. Standard Car

To facilitate the coding and classification system, we use the reference silhouettes to identify the type of car.

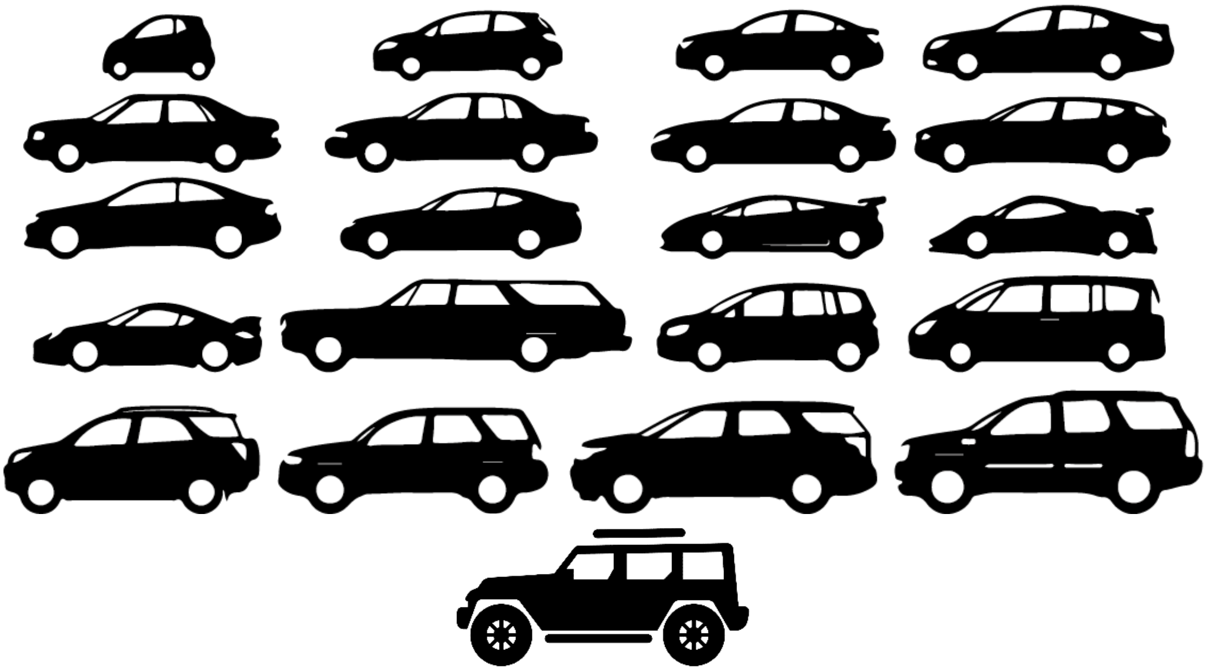

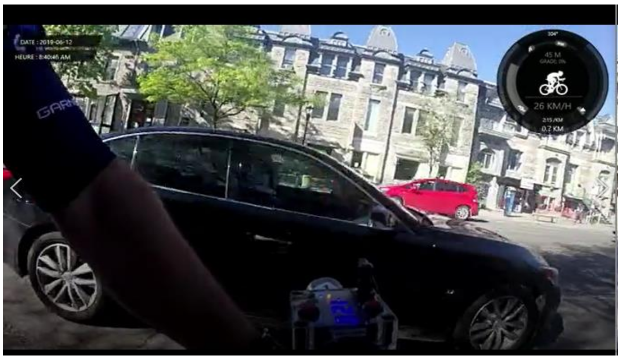

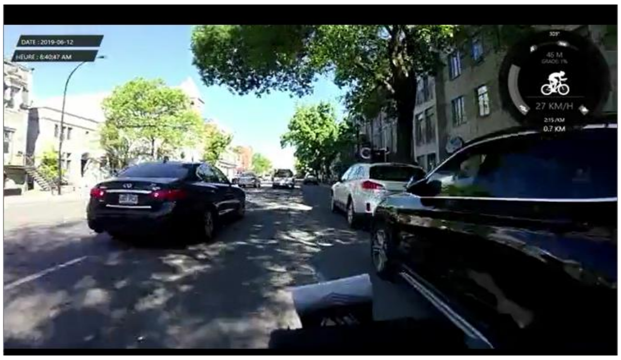

2.2. Pick-Up

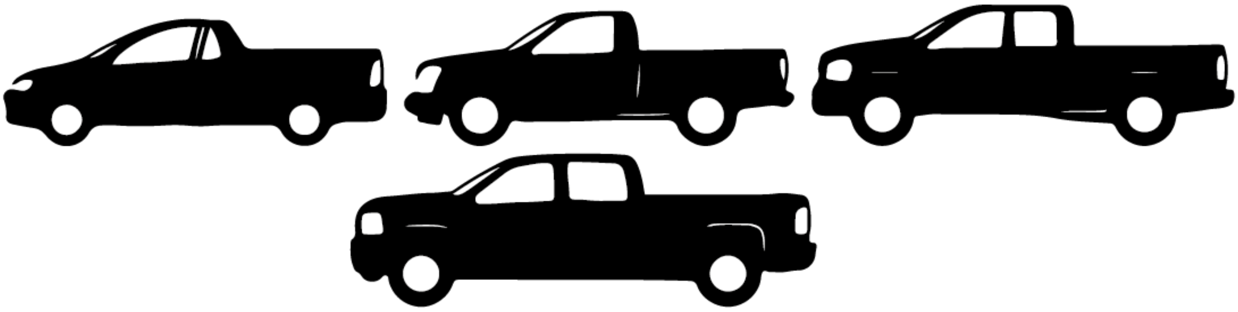

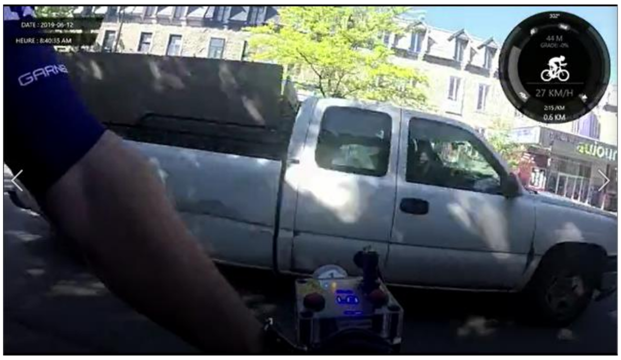

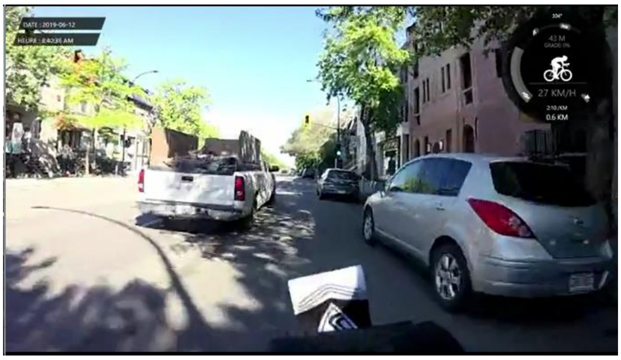

2.3. Truck

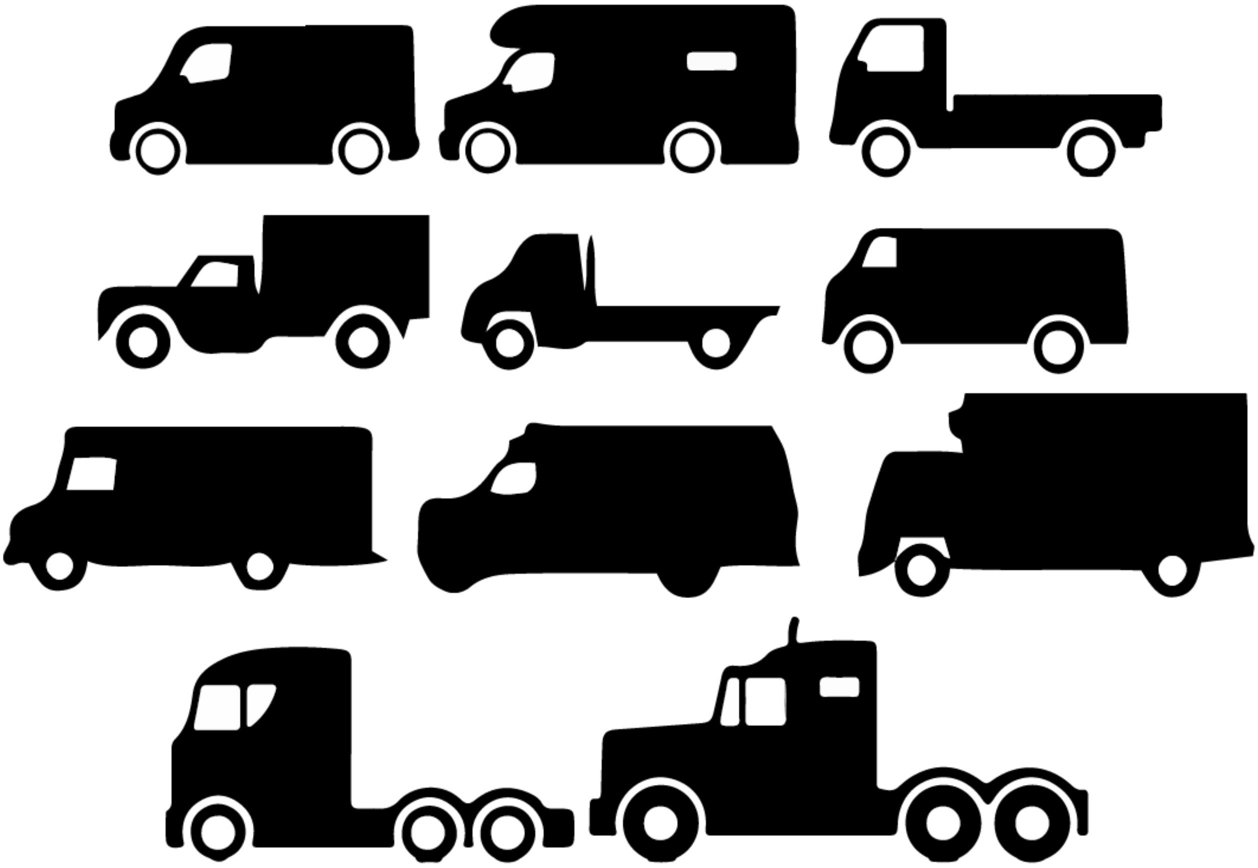

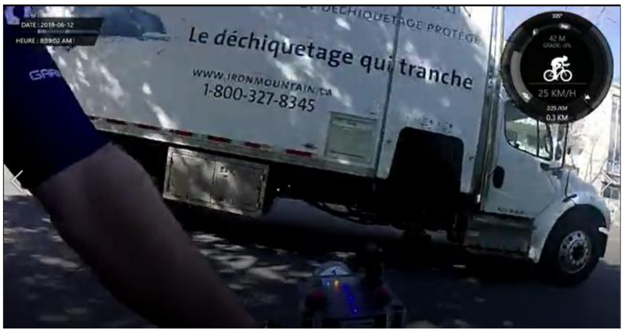
DATE : 2019-06-12
Le déchiquetage qui tranche
WWW.IRONMOUNTAIN.CA
1-800-327-8345
42 M
25 KM/H
0.3 KM

2.4. Bus

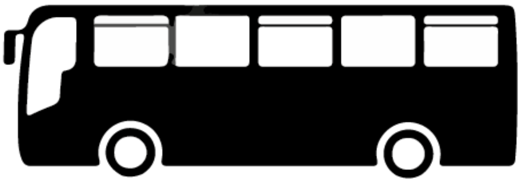

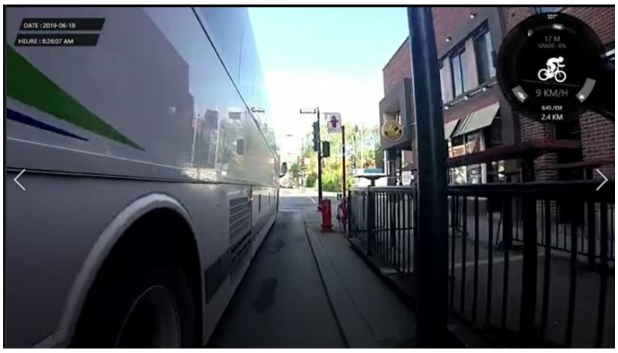

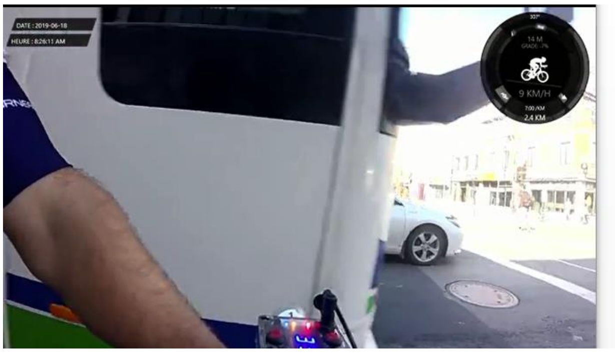

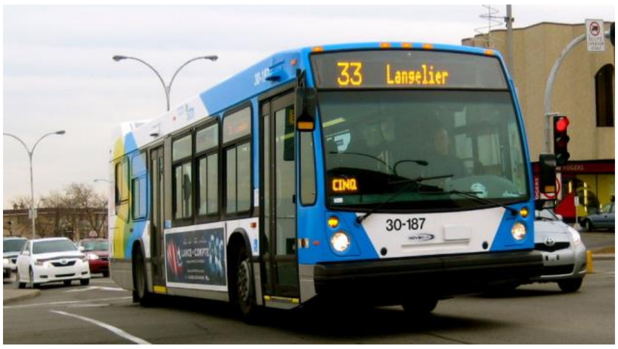

2.5. Other

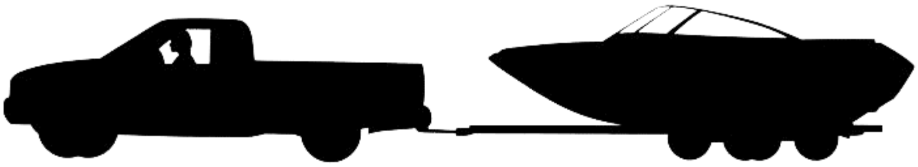

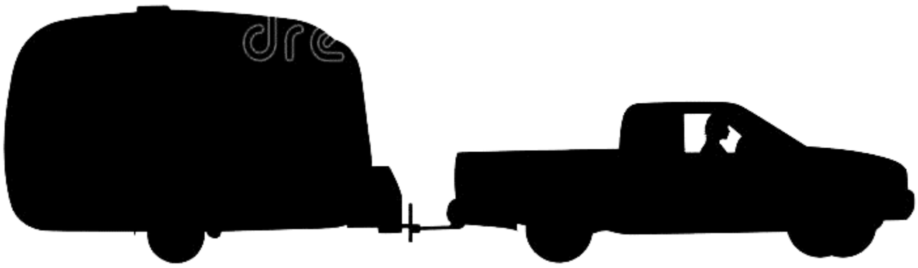

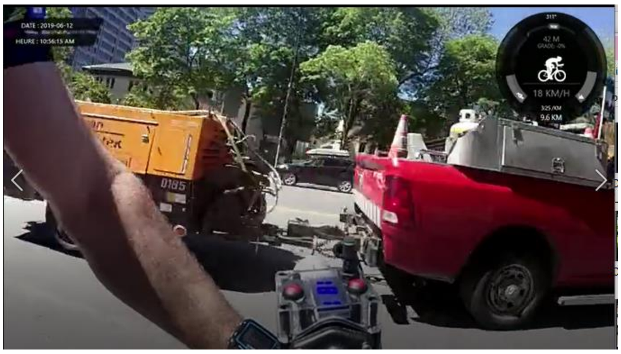

When an overtake is identified, the analyst should place an observation in Vifeco[1] at the start of the overtaking manoeuver (aligned to the handlebars) and at the end, as shown in the figure.

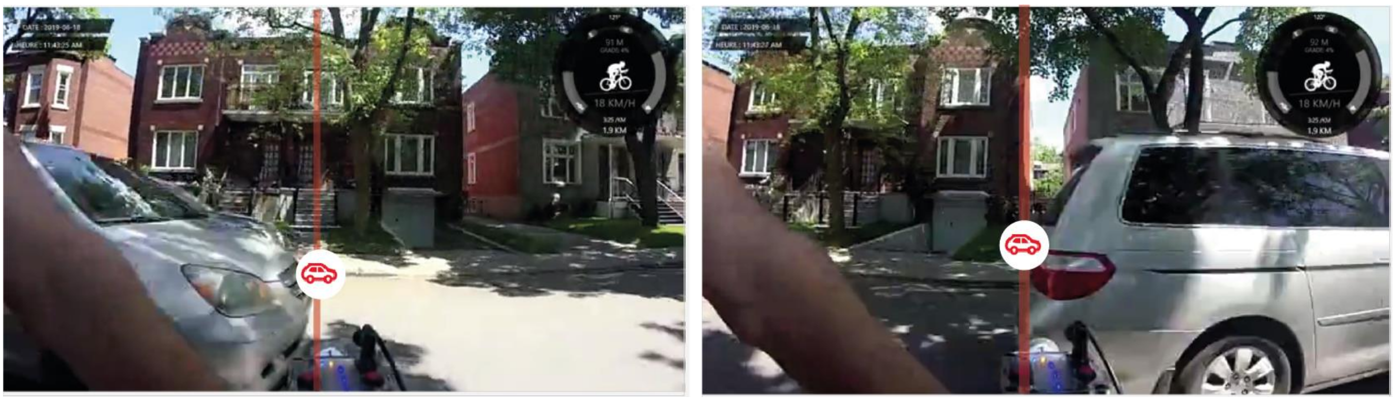

[1] an open-source software that allows to annotate several features on videos (link)

<table>
<tr><th colspan="4">Summary of the Guide to Counting Overtaking Manoeuvres– Andrés Henao et Philippe Apparicio</th></tr>
<tr><td colspan="4">1. Identify whether there is an overtaking manoeuvre according to the defined criteria</td></tr>
<tr><td rowspan="2">2. Identify the type of vehicle passing the cyclist</td><td>Standard car
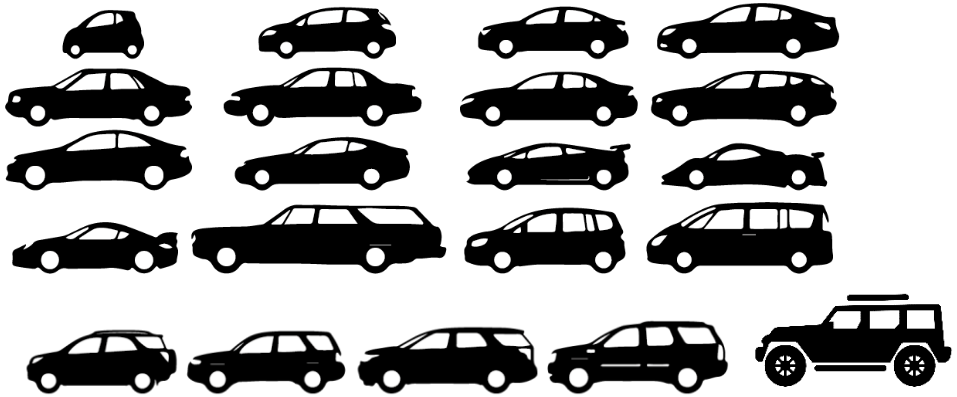</td><td colspan="2">Pick-up
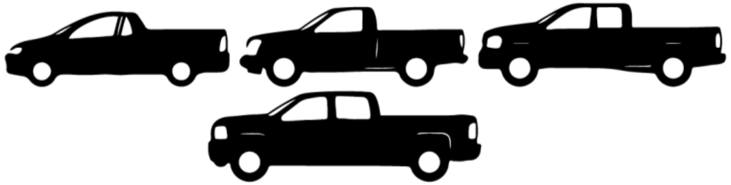</td></tr>
<tr><td>Truck
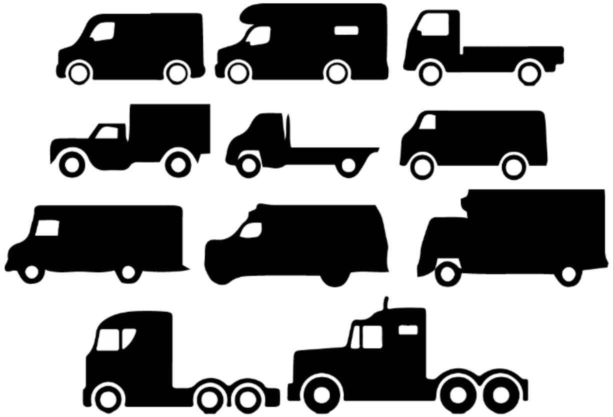</td><td>Bus
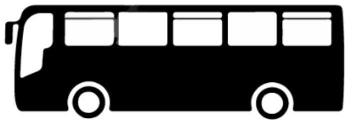</td><td>Other
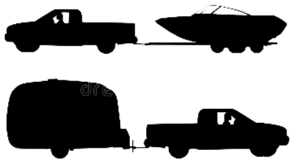</td></tr>
</table>
